# Solid-Phase Synthesis of *s*-Tetrazines

**DOI:** 10.1021/acs.orglett.3c00955

**Published:** 2023-04-21

**Authors:** Zainab S. Alghamdi, Maxime Klausen, Alessia Gambardella, Annamaria Lilienkampf, Mark Bradley

**Affiliations:** † EaStCHEM School of Chemistry, University of Edinburgh, Joseph Black Building, David Brewster Road, Edinburgh, EH9 3FJ, U.K.; ‡ Department of Chemistry, College of Science, Imam Abdulrahman Bin Faisal University, P.O. Box 1982, Dammam 31441, Saudi Arabia

## Abstract

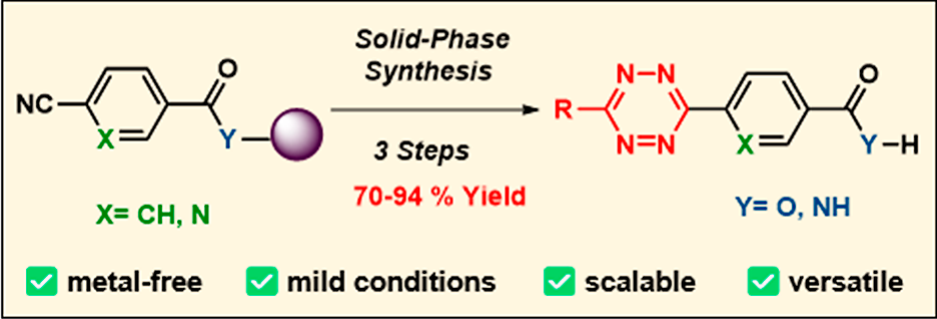

An efficient synthesis of *s*-tetrazines by solid-phase methods is described. This synthesis route was compatible with different solid-phase resins and linkers and did not require metal catalysts or high temperatures. Monosubstituted tetrazines were routinely synthesized using thiol-promoted chemistry, using dichloromethane as a carbon source, while disubstituted unsymmetrical aryl or alkyl tetrazines were synthesized using readily available nitriles. This efficient approach enabled the synthesis of *s*-tetrazines in high yields (70–94%), eliminating the classical solution-phase problems of mixtures of symmetrical and unsymmetrical tetrazines, with only a single final purification step required, and paves the way to the rapid synthesis of *s*-tetrazines with various applications in bioorthogonal chemistry and beyond.

*s*-Tetrazines (1,2,4,5-tetrazines) undergo inverse electron-demand Diels–Alder reactions with various dienophiles. They are powerful bioorthogonal cycloaddition reactions due to the rapid reactivity of tetrazines, nontoxic by-products (N_2_), and high reaction selectivity.^1−3^ As such, inverse electron-demand Diels–Alder reactions with tetrazines have been used in various biological scenarios such as sensing,^4^ imaging,^5,6^ and drug delivery.^7−9^ They have also been extended to applications in coordination chemistry,^10^ material science,^11^ and natural product synthesis.^12^ The clinical potential of bioorthogonal reactions involving tetrazines has been demonstrated with a first in-human inverse electron-demand Diels–Alder cycloaddition between a tetrazine decorated polymer and a *trans*-cyclooctene protected prodrug of Doxorubicin, allowing drug release at the site of the tumor where the polymer was implanted.^13,14^ A variety of mono- or disubstituted aliphatic^15−17^ and aromatic^18−20^ tetrazines have been used in bioorthogonal reactions, with monosubstituted tetrazines preferred due to greater reactivity, in part due to their small size. Tetrazines also have intrinsic fluorescence (λ_ex_/λ_em_ = 520–570 nm) with reasonable quantum yields (up to 0.44) and long fluorescence lifetimes (up to 180 ns).^21−23^ They have also been applied as “absorbers” in FRET pairs.^24,25^ Despite the ever-growing applications of tetrazines, their use has been hampered by laborious synthesis and purification and it is therefore important to develop new synthetic routes that provide easy access to substituted tetrazines with different reactivities. Many routes to aromatic/aliphatic *s*-tetrazines have been investigated, and the area has been well-reviewed.^26,27^ Conventional approaches toward *s*-tetrazines include a two-step synthesis starting from the condensation of hydrazine with aromatic nitrile precursors, followed by oxidation of the resulting 1,2-dihydrotetrazine to the tetrazine (Scheme 1).^28^ However, this approach is not suitable for aliphatic or unsymmetrical tetrazines, which are commonly prepared from aromatic or alkyl nitrile precursors and formamidine salts in low yields (<20%) and require several purification steps.^29^ Devaraj^30^ developed an efficient Lewis acid catalyzed (5 mol % of Zn(II) or Ni(II) salts) method for the synthesis of 3-substituted unsymmetrical *s*-tetrazines (30–70% yield), but this approach requires a large excess of potentially hazardous anhydrous hydrazine (50 equiv). Audebert^31^ reported a metal-free approach to monosubstituted tetrazines (40–70% yield), using hydrazine hydrate, sulfur, and dichloromethane (DCM) that acts as the source for the C-3 carbon within the tetrazine ring (Scheme 1). This approach required prolonged microwave irradiation (24 h) and had a relatively limited substrate scope as dichloroethane and dibromomethane both failed to generate tetrazines. Wu^32^ reported a scalable and high yielding organocatalytic synthesis to unsymmetrical alkyl and aryl tetrazines (34–75% yield) using the reversible reaction between nitriles and a thiol activator/catalyst, such as 3-mercaptopropionic acid or *N*-acetyl-l-cysteine. These formed thioimidate esters *in situ* with subsequent nucleophilic attack by hydrazine leading to regeneration of the thiol and formation of an amidrazone, which then reacted with another equivalent of thioimidate ester to give, after oxidation, the tetrazine (Scheme 1). Recently, Fox^33^ developed a one-pot method for the synthesis of 3-thiomethyltetrazines from carboxylic esters, with the 3-thiomethyltetrazines used in thioether reduction or palladium-catalyzed cross-coupling chemistries to generate mono- and disubstituted aliphatic or aromatic tetrazines (60–80% yield). Here, we report an expedient solid-phase synthesis route to both monosubstituted and unsymmetrical disubstituted *s*-tetrazines, bearing different functional groups, based on the thiol-promoted reaction between supported aryl nitriles and hydrazine. This resin-supported approach to tetrazines uses mild conditions, readily available materials, and was compatible with a variety of resins and linkers routinely used in solid-phase synthesis (Scheme 1).

**Scheme 1:**
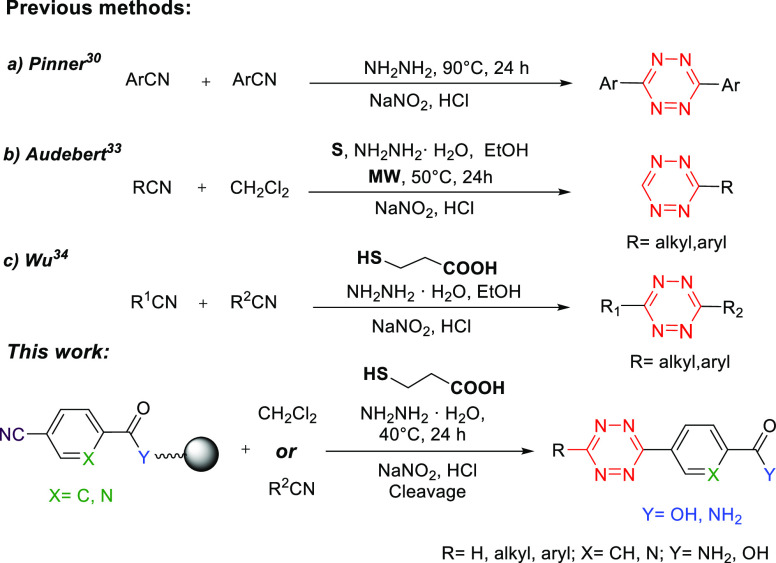
Previously Reported Methods to *s*-Tetrazines and Our Approach

As a proof-of-concept, thiol-promoted *s*-tetrazine synthesis^32^ on the solid-phase was investigated using a ChemMatrix resin (100 mg, loading 0.5–0.7 mmol/g, 100–200 mesh) functionalized with a Fmoc-Rink amide linker (Scheme 2). After *N*-Fmoc deprotection (20% piperidine), 4-cyanobenzoic acid (3 equiv) was coupled to the linker using DIC and Oxyma as the coupling combination. To form the monosubstituted 1,4-dihydrotetrazine **2**, the nitrile functionalized resin **1** was degassed and treated with hydrazine hydrate (0.06 M, 1 equiv) and 3-mercaptopropionic acid (3 equiv) in DCM (0.03 M), with the DCM here providing the C-3 carbon of the tetrazine as previously reported.^31^ The subsequent oxidation of **2** to the tetrazine was carried out, on-resin, using an aqueous solution of NaNO_2_ (0.1 M) with HCl (2 M). The resin beads turned deep pink within a few minutes confirming the formation of the monosubstituted, resin-bound tetrazine **3a** (Scheme 2). Cleavage off the solid support proved to be a crucial step in the synthesis, as tetrazines are prone to degradation under the strongly acidic conditions typically used to cleave acid-labile linkers in solid-phase synthesis (often 90% TFA is used for cleaving the Rink linker). The acid-mediated cleavage of tetrazine **3a** from the Rink linker was investigated looking at different reaction times and concentrations of TFA (Table 1). 90% TFA in H_2_O (entries 1–3) gave **4** in low yields (12–23%) with high levels of degradation and decreased yields due to prolonged exposure with TFA (shown visually by the change in color from pink to yellow and the low isolated yield after purification). Decreasing the acid concentration to 70% (entry 4) increased the yield significantly (45%), which increased to 71% (entry 5) after 3 × 1 h treatments without noticeable decomposition occurring. Similar yields (75%) were observed after 3 × 1 h treatments with 50% TFA. When water, which is traditionally used as a scavenger in the cleavage of the Rink linker, was removed from the “cleavage cocktail” (entries 7–9), the monosubstituted tetrazine **4** was isolated in 86% yield. Scaling up the reaction using 1 g of resin (with loadings of 0.6 or 1 mmol/g) did not affect the reaction, giving **4** in 88% and 90% yield, respectively (Table S1). The effect of using catalysts, such as sulfur or zinc triflate, was investigated; however, both gave **4** in lower yields (45% and 58%, respectively). This efficient solid-phase method was further expanded to the generation of disubstituted unsymmetrical *s*-tetrazines **5**–**10**, with a variety of aliphatic and aromatic nitriles (Scheme 2). Using the optimized conditions, the nitrile functionalized resin **1** was treated with hydrazine hydrate (0.06 M), degassed 3-mercaptopropionic acid (3 equiv), and the selected nitrile, either neat or dissolved in 1,4-dioxane. Note, it was crucial to eliminate any traces of DCM (typically used to swell the resin in solid-phase synthesis) in order to prevent preferential formation of monosubstituted tetrazine **4**. This synthetic approach was compatible with nitriles bearing both electron-donating (*e.g.*, methoxy) and electron-withdrawing groups (*e.g.*, nitro). The potential formation of undesired symmetrical disubstituted *s*-tetrazine side product(s) typically found in solution-phase synthesis is avoided, due to site isolation on the solid phase and the fact that any side products formed in solution are simply washed away. *tert*-Butylcyanoacetate gave access to carboxy-functionalized tetrazine **6** with the *tert-*butyl group removed during the acidic cleavage from the resin, although this compound proved to be poorly soluble. The Rink linker is widely used in solid-phase synthesis; however, it leaves behind a primary amide group after cleavage. Since tetrazines are commonly used in bioconjugation reactions, we expanded this methodology to a linker that would provide a “conjugation handle”, such as a carboxylic acid, upon cleavage, while also expanding the choice of resin being used. Thus, a 2-chlorotrityl chloride linker (CLTR-Cl) attached to a polystyrene resin was explored (Scheme 3). The scope of aryl nitriles was expanded using either 4-cyanobenzoic acid or 6-cyanonicotinic acids, which were attached to the trityl linker (loading 0.95 mmol/g) by esterification. Mono- and disubstituted carboxy-functionalized phenyl tetrazines **13**–**15**, pyridyl tetrazines **16**–**18**, and dipyridyl tetrazines **19**–**21** were formed using the same synthetic steps described above but with cleavage off the 2-chlorotrityl linker possible with 20% hexafluoroisopropanol (HFIP) in DCM, giving the tetrazines in excellent 78–94% yields. To explore the efficiency of our method to provide access to *ortho*- and *meta*-substituted *s*-tetrazines, 4-bromo- and 5-bromo-3-cyanopyridine were reacted via hydrazine condensation with the nitrile functionalized resin **11**. As expected, the *ortho*-functionalized *s*-dipyridyl tetrazine was not accessible owing to steric and electronic limitations,^34^ while the *meta-*functionalized *s*-dipyridyl tetrazine **19** was successfully synthesized in 85% yield. Moreover, considering the limited stability of the tetrazines, which is a known obstacle to their use, the tetrazines **4**–**21** reported here were observed to be robust for over 1–3 months on the solid support (storage in the dark at −20 °C).

**Scheme 2:**
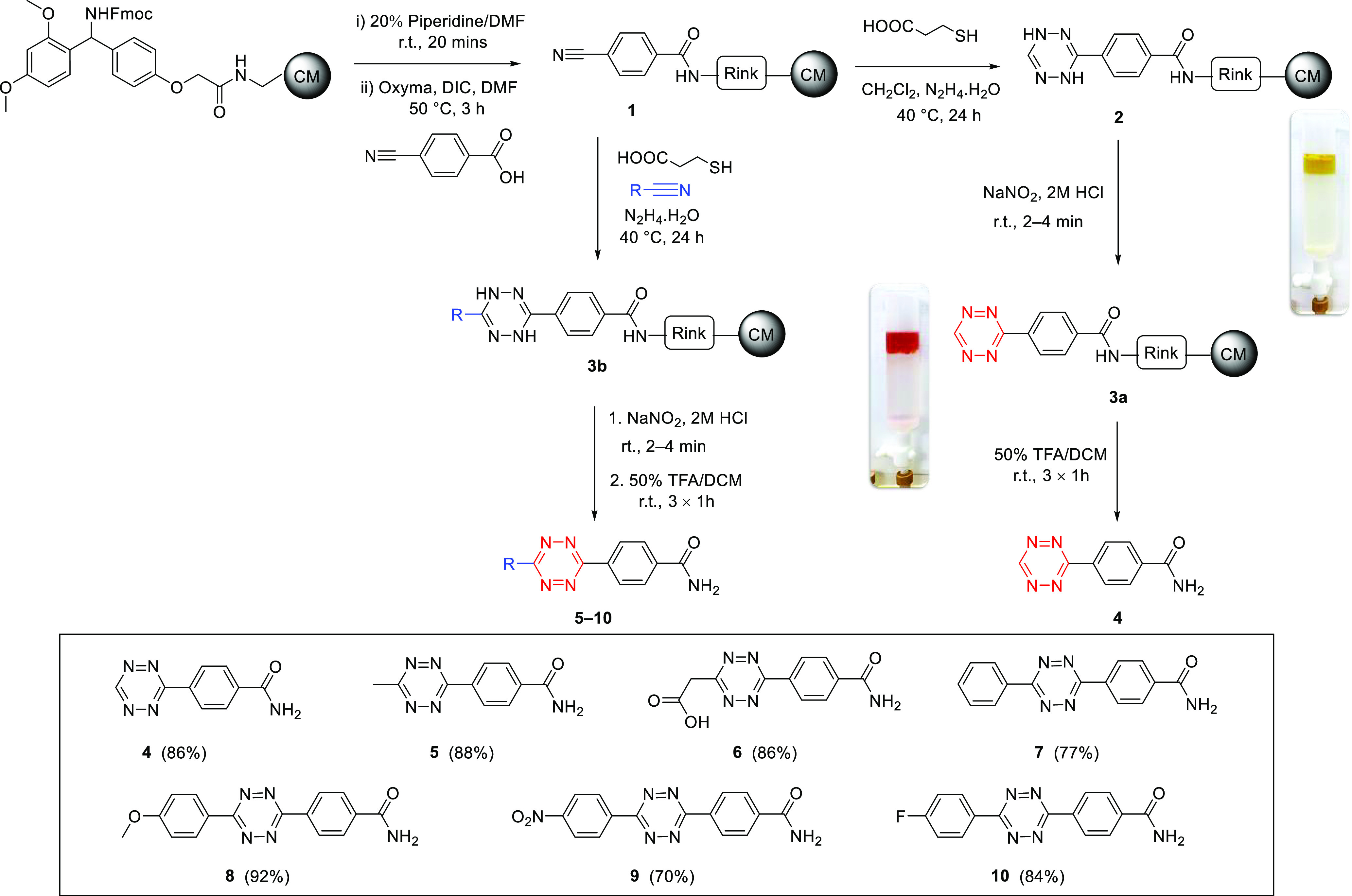
Solid-Phase Synthesis of Tetrazines Using a ChemMatrix (CM) Resin Functionalized with a Rink Amide Linker Illustrating the Scope of the Reaction The inset shows the structures and yields of the isolated products after purification.

**Table 1:**
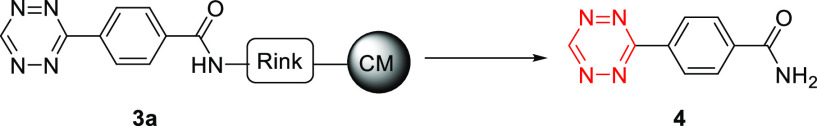
Optimization of the Resin Cleavage Conditions for the Efficient Liberation of the Tetrazines^a^

**Entry**	**Cleavage Cocktail**	**Time**	**Yield**^b^
**1**	90% TFA/H_2_O	3 h	12%
**2**	90% TFA/H_2_O	2 h	15%
**3**	90% TFA/H_2_O	1 h	23%
**4**	70% TFA/H_2_O	1 h	45%
**5**	70% TFA/H_2_O	3 × 1 h	71%
**6**	50% TFA/H_2_O	3 × 1 h	75%
**7**	50% TFA/DCM	1 h	49%
**8**	50% TFA/DCM	2 × 1 h	80%
**9**	50% TFA/DCM	3 × 1 h	86%

a Cleavage was performed at room temperature on 100 mg of ChemMatrix resin that had been preswollen in DCM, and the crude product was isolated by filtration and concentration.

b Isolated yield after purification by column chromatography.

**Scheme 3:**
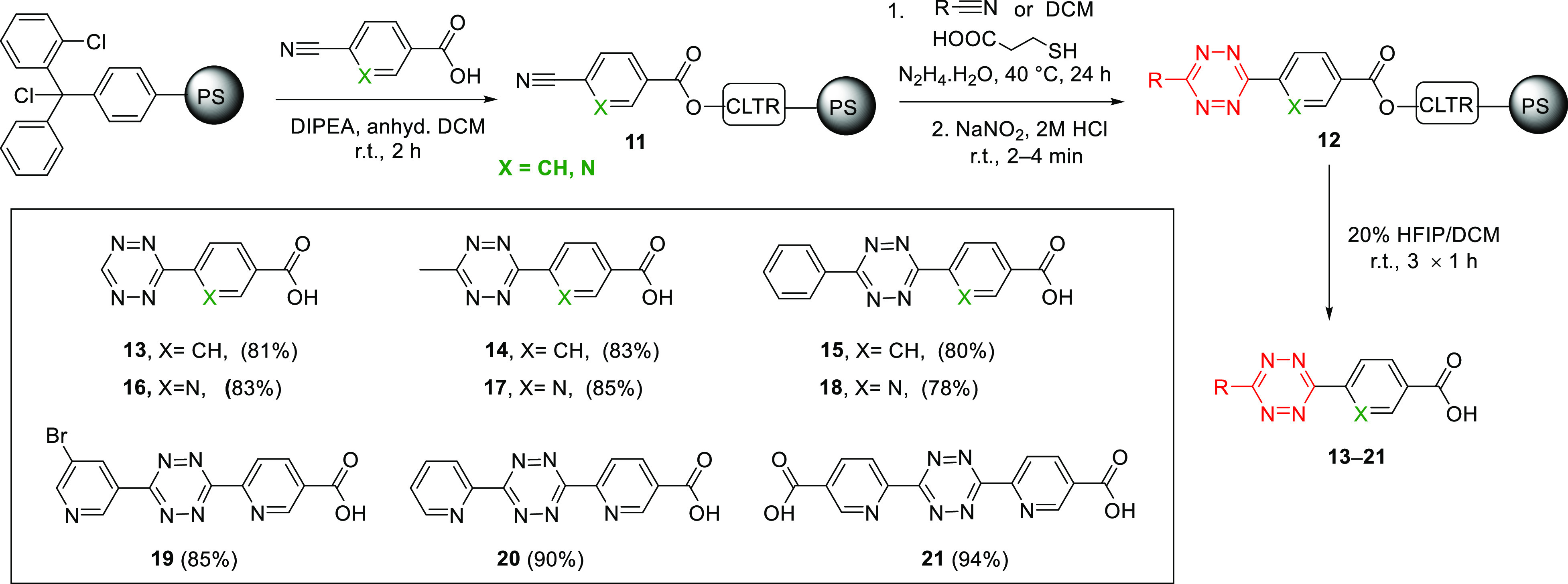
Solid-Phase Synthetic Route for Tetrazines on a 2-Chlorotritylchloride Linker Functionalized Polystyrene Resin Illustrating the Scope of the Reaction The inset shows the structures and yields of the isolated products obtained after purification.

In conclusion, a practical method for the synthesis of *s*-tetrazines has been developed, with the thiol-promoted pathway yielding mono- or disubstituted tetrazines. The methodology was compatible with different resin-supported aryl nitriles and aliphatic and aromatic acceptor nitriles with either electron-withdrawing or electron-donating groups. The method was versatile, using either DCM as the carbon source for monosubstituted tetrazines or nitriles (either as a solvent or as a reactant (in dioxane) for the disubstituted derivatives. All tetrazines were synthesized in excellent yields (70–94%) without the need for metal catalysts or high temperatures and notably required only a single purification step after cleavage. This solid-phase approach naturally overcomes the problems typically associated with disubstituted tetrazine synthesis in solution, namely, the formation and separation of the undesired symmetrical, disubstituted adducts. The method was compatible with different types of resins and linkers typically used in solid-phase synthesis. This route paves the way for applications in chemical biology where tetrazines can be synthesized *in situ* attached to peptides, thus providing a range of chemical handles that can be exploited in bioorthogonal chemistries, and also opens up routes to “on-resin” cyclization reactions.

## Data Availability

The data underlying this study are available in the published article and its Supporting Information.

## Supporting Information Available

Experimental procedures and characterization of compounds (PDF)
