# Supplementary material for: Solid-Phase Synthesis of s-Tetrazines
Source: Org Lett. 2023 Apr 21;25(17):3104–8. doi: 10.1021/acs.orglett.3c00955 (PMC10167685; doi:10.1021/acs.orglett.3c00955)
Supplement: Supplementary file 1 — ol3c00955_si_001.pdf [file ol3c00955_si_001.pdf]

Solid-Phase Synthesis of *s*-Tetrazines

Zainab Alghamdi,[a,b] Maxime Klausen,[a] Alessia Gambardella[a], Annamaria Lilienkampf[a] and Mark Bradley[a]

a. EaStCHEM School of Chemistry, University of Edinburgh, David Brewster Road, EH9 3FJ Edinburgh, UK.
b. Department of Chemistry, College of Science, Imam Abdulrahman Bin Faisal University, P.O. Box 1982, Dammam 31441, Saudi Arabia.

Table of Contents

1. Experimental 2

1.1. Materials and Methods 2

2. General solid-phase synthesis methods for amide tetrazines **4**–**10**..........................3

3. General solid-phase synthesis methods for carboxyl tetrazines **13**–**21**.......................9

4. ^{1}H and ^{13}C NMR Spectra..17

5. HRMS...32

6. References..40

1. Experimental

1.1. Materials and Methods

Aminomethyl ChemMatrix resin (0.6 mmol/g, 100–200 mesh, 1% DVB), 2-chlorotrityl chloride polystyrene resin (0.945 mmol/g, 100–200 mesh, 1% DVB), Fmoc-Rink Amide-Linker, and all other chemicals were purchased from GL Biochem, Fluorochem, Apollo Scientific, Sigma Aldrich or Acros. Commercially available reagents were used without further purification.

^{1}H and ^{13}C NMR spectra were recorded on an automated Bruker AVA 500 (at 500 and 126 MHz, respectively) or Bruker AVA 600 (at 600 and 151 MHz, respectively). Chemical shifts are reported on the δ scale with respect to the non-deuterated solvent residual peak for ^{1}H spectra and the deuterated carbon resonance for ^{13}C. All coupling constants (*J*) were measured in Hertz (Hz). Resonances are labelled as singlet (s), doublet (d), triplet (t), and multiplet (m). Low resolution electrospray ionization mass spectrometry (ESI-MS) analyses were carried out on an Agilent Technologies LC/MSD Series 1100 quadrupole mass spectrometer (QMS) in ESI mode. HR-MS were measured on a Finnigan MAT 900 XLP high resolution double-focusing mass spectrometer. Fourier transform ion cyclotron resonance mass spectrometry. High-resolution mass spectra were recorded on a 12T Bruker Daltonics-Fourier transform ion cyclotron resonance mass spectrometer (FT-ICR-MS) using electrospray ionisation at 4.5 kV. The spectra were acquired with a m/z range of 147 to 3000, and a minimal collisional activation of -2 V was used. The ions accumulation was set at 200 ms, and 16 Free Induced Decay (FID) transients were combined to produce a mass spectrum at 2 MW resolution.

Flash column chromatography was performed using silica gel 60 (230–400 mesh, Merck) with a forced flow of eluent. Preparative reverse phase HPLC (RP–HPLC) was performed on an Agilent 1100 system equipped with a Kinetex XB-C18, AXIA Packed reverse-phase column (150 x 21.2 mm, 5 µm, 100A) with a flow rate 10 mL/min, eluting with 0.1% HCOOH in H_2O (A) and 0.1% HCOOH in CH_3CN (B), with a gradient of 5 to 95% B over 18 min and additional isocratic period of 1 min.

Analytical reverse-phase high-performance liquid chromatography (RP-HPLC) was performed on an Agilent 1100 system equipped with a Kinetex XB-C18 column (50 × 4.6 mm, 5 µm) with a flow rate of 1 mL/min. Samples were eluted with a gradient of H_2O/MeCN 95/5 to 5/95, buffered with 0.1% formic acid, over 6 min, then holding at 95% for 3 min, followed by elution at 5% MeCN. Detection was performed with a multiple wavelength detector (MWD) at 254, 282 and 495 nm, and by an evaporative light scattering (ELSD) detector.

2. General solid-phase synthesis methods for amide tetrazines 4–10

Rink-amide linker attachment to Aminomethyl ChemMatrix resin. 4-[(2,4-Dimethoxyphenyl) -(Fmoc-amino)methyl]phenoxyacetic acid (Fmoc-Rink amide-linker, 0.97 g, 1.8 mmol, 3 equiv.) and ethyl(hydroxyimino)cyanoacetate (Oxyma, 0.26 g, 1.8 mmol, 3 equiv.) were dissolved in DMF (5 mL, 0.1 M) and the mixture was stirred for 10 min. *N,N′*-Diisopropylcarbodiimide (DIC, 279 µL, 1.8 mmol, 3 equiv.) was added and the solution was stirred for 1 min. This solution was added to the resin (1.0 g, 0.6 mmol/g, 1 equiv., pre-swollen in DCM) and the mixture was stirred at 50 °C in a DMF bath for 1 h. After cooling down to room temperature, the resin was drained and washed with DMF (3 × 10 mL), DCM (3 × 10 mL) and MeOH (3 × 10 mL). The coupling reaction was monitored by a Kaiser test.[1] To cap any remaining free amines, the resin was treated with a mixture of Ac_2O:Pyridine:DMF (2:3:15, *v/v/v)* for 30 min, followed by washing with DMF (3 × 10 mL), DCM (3 × 10 mL), MeOH (3 × 10 mL) and Et_2O (10 mL).

Fmoc deprotection. A solution of 20% piperidine in DMF (5 mL, 0.1 M) was added to the resin (1.0 g, 0.6 mmol/g, pre-swollen in DCM), and the reaction mixture was shaken for 10 min. The solution was then drained and the resin was washed with DMF (3 × 10 mL), DCM (3 × 10 mL) and MeOH (3 × 10 mL). This procedure was repeated twice.

Cyanobenzoic acid coupling. The appropriate cyanobenzoic acid (0.27 g, 1.8 mmol, 3 equiv.) and Oxyma (0.26 g, 1.8 mmol, 3 equiv.) were dissolved in DMF (5 mL, 0.1 M) and stirred for 10 min. DIC (1.8 mmol, 3 equiv.) was added and the solution was stirred for 1 min. The mixture was then added to the resin (1.0 g, 0.6 mmol/g, pre-swollen in DCM), and the reaction mixture

was stirred at 50 °C in a DMF bath for 2 h. The resin was drained and washed with DMF (3 × 10 mL), DCM (3 × 10 mL) and MeOH (3 × 10 mL). The coupling reaction was monitored by the Kaiser test.

Dihydrotetrazine formation. A solution of 3-mercaptopropionic acid (15.7 μL, 0.18 mmol, 3 equiv.) and hydrazine hydrate (1 mL, 0.06 M) was added to the benzonitrile functionalized Rink resin (100 mg, 60 μmol, 1 equiv.) with either DCM or nitrile precursors to form either the mono- or disubstituted dihydrotetrazine with variety of functional groups. Further details on the scope of the reaction are given on page S5.

Oxidation of dihydrotetrazines to tetrazines. A solution of $NaNO_2$ (70 mg, 1.0 mmol, 17 equiv.) in water (5 mL, 0.1 M) was added to the resin (100 mg, 60 μmol/g, 1 equiv., pre-swollen in DCM), followed by dropwise addition of aqueous HCl (2 M) until pH ~3 was obtained. The resin beads turned pink-red within 2–4 minutes indicating the formation of the tetrazine (Caution: this procedure must be carried out in a well-ventilated fume hood due to the formation of nitrous fumes at this stage). The resin was drained and washed with DMF (3 × 10 mL), DCM (3 × 10 mL) and MeOH (3 × 10 mL).

Cleavage off the resin. The resin (100 mg, 60 μmol, 0.1 equiv., pre-swollen in DCM) was shaken for 3 h in a mixture of TFA/DCM (1 mL, 1:1 *v/v*). The solution was collected by filtration and the resin was rinsed with a portion of the cleavage mixture (0.5 mL). The combined filtrates were diluted with toluene (5 mL) to avoid the degradation of tetrazine due to TFA, when the solvents were evaporated to dryness *in vacuo*.

4-(1,2,4,5-Tetrazin-3-yl)benzamide (4)

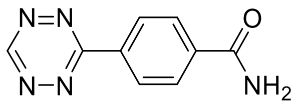

A mixture of DCM (2 mL, 0.03 M), 3-mercaptopropionic acid (15.7 μL, 0.18 mmol, 3 equiv.), and hydrazine hydrate (1 mL, 0.06 M) was added to the benzonitrile functionalized Rink resin (100 mg, 60 μmol, 1 equiv., pre-swollen in DCM). The reaction mixture was stirred in a sealed vial at 40 °C in a heating mantle for 24 h. The resin was drained and washed with DMF (3 × 10

mL), DCM (3 × 10 mL) and MeOH (3 × 10 mL). After oxidation and cleavage, the crude product was purified by column chromatography (Petroleum ether/EtOAc/AcOH 50:50:1, *v/v/v*) to give compound **4** as a pink solid (10 mg, 86%).

^{1}H NMR (500 MHz, DMSO-d_6) δ 10.64 (s, 1H), 8.57 (d, *J* = 8.5 Hz, 2H), 8.19 (s, 1H), 8.14 (d, *J* = 8.5 Hz, 2H), 7.58 (s, 1H). **^{13}C NMR** (126 MHz, DMSO-d_6) δ 167.1, 165.2, 158.2, 137.9, 134.3, 128.5, 127.7. **HRMS** (ESI) for $C_9H_7O_1N_5Na_1$ [M+Na]$^+$ *calcd*. 224.0543; *found* 224.0534.

4-(6-Methyl-1,2,4,5-tetrazin-3-yl)benzamide (5)

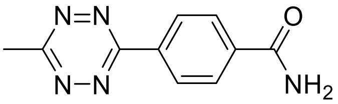

A mixture of acetonitrile (2 mL, 0.03 M), 3-mercaptopropionic acid (15.7 μL, 0.18 mmol, 3 equiv.), and hydrazine hydrate (1 mL, 0.06 M) was added to the benzonitrile functionalized resin (100 mg, 60 μmol, 1 equiv., pre-swollen in 1,4-dioxane). The reaction mixture was stirred in a sealed vial at 40 °C in a heating mantle for 24 h. The resin was drained and washed with DMF (3 × 10 mL), DCM (3 × 10 mL) and MeOH (3 × 10 mL). After oxidation and cleavage, the crude product was purified by column chromatography (petroleum ether/EtOAc/AcOH = 50:50:1.5, *v/v/v*) to give compound **5** as a red/pink solid (12 mg, 88%).

^{1}H NMR (600 MHz, DMSO-d_6) δ 8.53 (d, *J* = 8.5 Hz, 2H), 8.18 (s, 1H), 8.13 (d, *J* = 8.5 Hz, 2H), 7.56 (s, 1H), 3.02 (s, 3H). **^{13}C NMR** (151 MHz, DMSO-d_6) δ 167.3, 167.2, 166.4, 162.9, 137.6, 134.3, 128.5, 127.3, 20.9. **HRMS** (ESI) for $C_{10}H_{10}N_5O_1$ [M+H]$^+$ *calcd*. 216.0880; *found* 216.0883.

2-(6-(4-carbamoylphenyl)-1,2,4,5-tetrazin-3-yl)acetic acid (6)

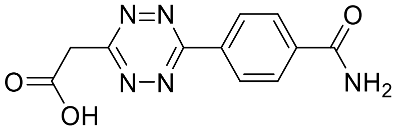

A mixture of *tert*-Butylcyanoacetate (2 mL, 0.03 M), 3-mercaptopropionic acid (15.7 μL, 0.18 mmol, 3 equiv.), and hydrazine hydrate (1 mL, 0.06 M) was added to the benzonitrile functionalized resin (100 mg, 60 μmol, 1 equiv., pre-swollen in 1,4-dioxane). The reaction mixture was stirred in a sealed vial at 40 °C in a heating mantle for 24 h. The resin was drained and washed with DMF (3 × 10 mL), DCM (3 × 10 mL) and MeOH (3 × 10 mL). After the following oxidation and cleavage, the resulting crude was washed and centrifuged with DMSO (3 × 1 mL), acetone (3 × 3 mL) then DCM (3 × 3 mL) and the supernatant solutions were decanted to give compound **6** as a purple solid (14 mg, 86%).

HRMS (ESI) for $C_{11}H_{10}N_5O_3$ $[M+H]^+$ *calcd.* 260.0778; *found* 260.0784.

Note: Due to the very poor solubility of compound 6 in all NMR solvents tried, NMR spectrum could not be recorded.

4-(6-Phenyl-1,2,4,5-tetrazin-3-yl)benzamide (7)

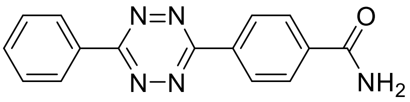

A mixture of benzonitrile (2 mL, 0.03 M), 3-mercaptopropionic acid (15.7 μL, 0.18 mmol, 3 equiv.), and hydrazine hydrate (1 mL, 0.06 M) was added to the benzonitrile functionalized resin (100 mg, 60 μmol, 1 equiv., pre-swollen in 1,4-dioxane). The reaction mixture was stirred in a sealed vial at 40 °C in a heating mantle for 24 h. The resin was drained and washed with DMF (3 × 10 mL), DCM (3 × 10 mL) and MeOH (3 × 10 mL). After the following oxidation and cleavage, the resulting crude was washed and centrifuged with DMSO (3 × 1 mL), then DCM (3 × 3 mL) and the supernatant solutions were decanted to give compound **7** as a purple solid (13 mg, 77%).

^{1}H NMR (500 MHz, DMF-*d_7*) δ 8.70 (d, *J* = 8.5, 2H), 8.65 (d, *J* = 8.5 Hz, 2H), 8.33 (s, 1H), 8.30 (d, *J* = 8.5 Hz, 2H), 7.82 – 7.72 (m, 3H), 7.58 (s, 1H). **^{13}C NMR** (126 MHz, DMF-*d_7*) δ 167.6, 164.1, 163.7, 138.3, 134.8, 132.9, 132.4, 129.6, 129.05, 128.7, 127.9. **HRMS** (ESI) for $C_{15}H_{12}N_5O_1$ $[M+H]^+$ *calcd*. 278.1036; *found* 278.1033.

4-(6-(4-Methoxyphenyl)-1,2,4,5-tetrazin-3-yl)benzamide (8)

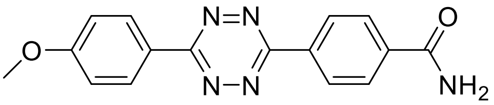

A solution of 4-methoxybenzonitrile (24 mg, 0.18 mmol, 3 equiv.) in 1,4-dioxane (2 mL, 0.03 M), 3-mercaptopropionic acid (15.7 μL, 0.18 mmol, 3 equiv.), and hydrazine hydrate (1 mL, 0.06 M) were added to the benzonitrile functionalized resin (100 mg, 60 μmol, 1 equiv., pre-swollen in 1,4-dioxane). The reaction mixture was stirred in a sealed vial at 40 °C in a heating mantle for 24 h. The resin was drained and washed with DMF (3 × 10 mL), DCM (3 × 10 mL) and MeOH (3 × 10 mL). After the following oxidation and cleavage, the resulting crude was washed with hexane (3 × 3 mL) and then MeOH (3 × 3 mL), with the product collected by centrifugation after each wash. Compound **8** was obtained as a pink solid (18 mg, 92%).

^{1}H NMR (600 MHz, DMF-*d$_7$*) δ 8.67 (d, *J* = 8.5 Hz, 2H), 8.61 (d, *J* = 8.5 Hz, 2H), 8.31 (s, 1H), 8.29 (d, *J* = 8.5 Hz, 2H), 7.57 (s, 1H), 7.31 (d, *J* = 8.5 Hz, 2H), 4.00 (s, 3H). **^{13}C NMR** (126 MHz, DMF-*d$_7$*) δ 168.6, 164.7, 164.2, 139.1, 135.9, 130.8, 129.6, 128.8, 128.4, 125.5, 116.1, 56.5. **HRMS** (ESI) for $C_{16}H_{14}N_5O_2$ $[M+H]^+$ *calcd*. 308.1142; *found* 308.1139.

4-(6-(4-Nitrophenyl)-1,2,4,5-tetrazin-3-yl)benzamide (9)

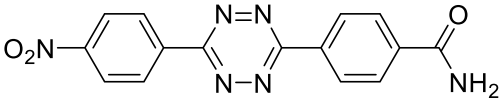

A solution of 4-nitrobenzonitrile (26.7 mg, 0.18 mmol, 3 equiv.) in 1,4-dioxane (2 mL, 0.03 M), 3-mercaptopropionic acid (15.7 μL, 0.18 mmol, 3 equiv.), and hydrazine hydrate (1 mL, 0.06 M) were added to benzonitrile functionalized resin (100 mg, 60 μmol, 1 equiv., pre-swollen in 1,4-dioxane). The reaction mixture was stirred in a sealed vial at 40 °C in a heating mantle for 24 h. The resin was drained and washed with DMF (3 × 10 mL), DCM (3 × 10 mL) and MeOH (3 × 10 mL). After the following oxidation and cleavage, the resulting crude was

washed and centrifuged with hexane (3 x 3 mL), then MeOH (3 × 3 mL) and the supernatant solutions were decanted to give compound **9** as a pink solid (14 mg, 70%).

*Note: Due to the low solubility of compound **9** in DMF-d_7 and other common NMR organic solvents, a ^{13}C NMR spectrum could not be recorded. Prior to HRMS (ESI) analysis, the IMS-MS was tuned and a mass calibration was performed using Agilent Tune Mix from the manufacturer (Cat No.: G2421-60001, Agilent Technologies).*

^{1}H NMR (500 MHz, DMF-*d_7*) δ 8.70 (d, *J* = 8.5 Hz, 2H), 8.65 (d, *J* = 8.5 Hz, 2H), 8.34 – 8.27 (m, 3H), 7.76 (d, 2H), 7.59 (s, 1H). **HRMS** (ESI) for of $C_{15}H_{11}N_6O_3$ [M+H]$^+$ *calcd*. 323.0887; *found* 323.0885.

*Note: Due to the low solubility of compound **9** in DMF-d_7 and other common NMR organic solvents, a ^{13}C NMR spectrum could not be recorded. Prior to HRMS (ESI) analysis, the IMS-MS was tuned and a mass calibration was performed using Agilent Tune Mix from the manufacturer (Cat No.: G2421-60001, Agilent Technologies).*

4-(6-(4-Fluorophenyl)-1,2,4,5-tetrazin-3-yl)benzamide (10)

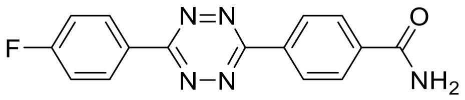

A solution of 4-fluorobenzonitrile (22 mg, 0.18 mmol, 3 equiv.) in 1,4-dioxane (2 mL, 0.03 M), 3-mercaptopropionic acid (15.7 μL, 0.18 mmol, 3 equiv.), and hydrazine hydrate (1 mL, 0.06 M) were added to the benzonitrile functionalized resin (100 mg, 60 μmol, 1 equiv., pre-swollen in 1,4-dioxane). The reaction mixture was stirred in a sealed vial at 40 °C in a heating mantle for 24 h. The resin was drained and washed with DMF (3 × 10 mL), DCM (3 × 10 mL) and MeOH (3 × 10 mL). After the following oxidation and cleavage, the resulting crude was washed and centrifuged with hexane (3 × 3 mL), and DCM (3 × 3 mL) then EtOH (3 × 3 mL) and the supernatant solutions were decanted to give compound **10** as a pink solid (15 mg, 84%).

^{1}H NMR (500 MHz, DMF-*d_7*) δ 8.72 (d, *J* = 8.5 Hz, 2H), 8.70 (d, *J* = 8.5 Hz, 2H), 8.33 (s, 1H), 8.30 (d, *J* = 8.5 Hz, 2H), 7.61 (s, 1H), 7.58 (d, *J* = 8.5 Hz, 2H). **^{13}C NMR** (126 MHz, DMF-*d_7*) δ 168.6,

167.9, 167.7, 164.6, 139.3, 135.7, 133.5, 131.6, 131.5 (d, *J* = 9.4 Hz), 129.7, 129.5, 128.7, 117.8, 117.6 (d, *J* = 22.4 Hz). **HRMS** (ESI) for $C_{15}H_{10}F_1N_5O_1K_1$ $[M+K]^+$ *calcd*. 334.0501; *found* 334.0500.

3. General solid-phase synthesis methods for carboxyl tetrazines 13–21

Activation of 2-chloro/hydroxy-trityl chloride linker (CLTR-Cl) polystyrene resin. A solution of $SOCl_2$ (240 µL, 1.2 mmol, 1.3 equiv.) in anhydrous DCM (10 mL, 0.1 M) was added to the commercially available 2-chloro/hydroxy-trityl chloride linker polystyrene resin (1.0 g, 0.95 mmol/g, 1 equiv., pre-swollen in anhydrous DCM) under an Ar atmosphere. The reaction mixture was stirred for 1 h. The solvent was drained and the resin was washed with anhydrous DCM (3 × 10 mL) and anhydrous DMF (3 × 10 mL) and used immediately. Note: This activation process converts any hydrolyzed sites to the chloride before subsequent esterification.

Attachment of benzoic or nicotinic acid on the linker. A solution of either benzoic or nicotinic acid (2.5 mmol, 3 equiv., 0.2 M) and DIPEA (456 µL, 4.8 mmol, 5 equiv.) in anhydrous DCM/DMF (7:1, *v/v*) was added to the pre-activated resin (1.0 g, 0.95 mmol/g, pre-swollen in anhydrous DCM), and the reaction mixture was stirred for 1 h. The solution was drained and the resin was washed with anhydrous DCM (3 × 10 mL) and anhydrous DMF (3 × 10 mL). To cap any unreacted linker, the resin was treated with a mixture of DCM/MeOH/DIPEA (8:1.5:0.5, *v/v/v*) for 2 × 15 min followed by washing with DMF (3 × 10 mL), ^{i}PrOH (3 × 10 mL) and hexane (3 × 10 mL).

Dihydrotetrazine formation. A solution of 3-mercaptopropionic acid (25.3 µL, 0.29 mmol, 3 equiv.) and hydrazine hydrate (1 mL, 0.1 M) was added to the nitrile functionalized trityl resin (100 mg, 95 µmol, 1 equiv.) with either DCM or nitrile precursors to form either the mono- or disubstituted dihyrdrotetrazines. Further details on the scope of the reaction are given on page S10.

Oxidation of dihydrotetrazines to tetrazines. A solution of $NaNO_2$ (70 mg, 1.0 mmol, 10 equiv.) in water (5 mL, 0.1 M) was added to the resin (100 mg, 95 µmol/g, 1 equiv., pre-swollen in DCM), followed by dropwise addition of 2 M HCl (aq.) until pH ~3 was obtained.

The resin beads turned pink-red within 2–4 min indicating the formation of a tetrazine (Caution: this procedure must be carried out in a well-ventilated fume hood due to the formation of nitrous fumes at this stage).

Cleavage off the resin. The resin (100 mg, 95 μmol, 1 equiv., pre-swollen in DCM) was shaken for 2 h in 20% HFIP in DCM (1 mL). The solution was collected by filtration and the resin was rinsed with 20% HFIP in DCM (0.5 mL). The solutions were combined and evaporated to dryness *in vacuo*.

4-(1,2,4,5-Tetrazin-3-yl)benzoic acid (13)

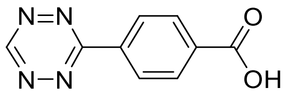

A mixture of DCM (2 mL, 0.05 M), 3-mercaptopropionic acid (25.3 μL, 0.29 mmol, 3 equiv.), and hydrazine hydrate (1 mL, 0.1 M) was added to the nitrile functionalized trityl linker-resin (100 mg, 95 μmol, 1 equiv., pre-swollen in DCM). The reaction mixture was stirred in a sealed vial at 40 °C in a heating mantle for 24 h. The resin was drained and washed with DMF (3 × 10 mL), DCM (3 × 10 mL) and MeOH (3 × 10 mL). After oxidation and cleavage, the crude product was purified by column chromatography (Petroleum ether/EtOAc/AcoH 50:50:1, *v/v/v*) to give compound **13** as a pink solid (16 mg, 81%). Analytical data in agreement with literature.[2]

^{1}H NMR (600 MHz, DMSO-d_6) δ 10.66 (s, 1H), 8.61 (d, *J* = 8.4 Hz, 2H), 8.22 (d, *J* = 8.4 Hz, 2H). **^{13}C NMR** (126 MHz, DMSO-d_6) δ 166.7, 165.1, 158.3, 135.7, 132.7, 130.2, 128.0. **HRMS** (ESI) for $C_9H_5N_4O_2$ $[M-H]^-$ *calcd*. 201.0418; *found* 201.0408.

4-(6-Methyl-1,2,4,5-tetrazin-3-yl)benzoic acid (14)

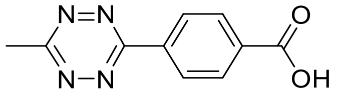

A mixture of acetonitrile (2 mL, 0.05 M), 3-mercaptopropionic acid (25.3 μL, 0.29 mmol, 3 equiv.), and hydrazine hydrate (1 mL, 0.1 M) was added to the nitrile functionalized trityl

linker-resin (100 mg, 95 μmol, 1 equiv., pre-swollen in 1,4-dioxane). The reaction mixture was stirred in a sealed vial at 40 °C in a heating mantle for 24 h. The resin was drained and washed with DMF (3 × 10 mL), DCM (3 × 10 mL) and MeOH (3 × 10 mL). After oxidation and cleavage, the crude product was purified by column chromatography (Petroleum ether/EtOAc/AcOH 50:50:1.5, *v/v/v*) to give compound **14** as a pink solid (17 mg, 83%). Analytical data in agreement with literature.[2]

^{1}H NMR (600 MHz, DMSO-*d$_6$*) δ 8.58 (d, *J* = 8.4 Hz, 2H), 8.20 (d, *J* = 8.4 Hz, 2H), 3.03 (s, 3H). **^{13}C NMR** (151 MHz, DMSO-*d$_6$*) δ 167.4, 166.7, 162.8, 135.7, 134.0, 130.2, 127.6, 20.9. **HRMS** (ESI) for $C_{10}H_9N_4O_2$ $[M+H]^+$ *calcd*. 217.0720; *found* 217.0722.

4-(6-Phenyl-1,2,4,5-tetrazin-3-yl)benzoic acid (15)

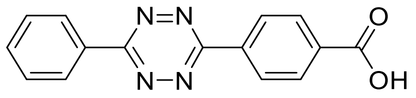

A mixture of benzonitrile (2 mL, 0.05 M), 3-mercaptopropionic acid (25.3 μL, 0.29 mmol, 3 equiv.), and hydrazine hydrate (1 mL, 0.1 M) was added to the nitrile functionalized trityl linker-resin (100 mg, 95 μmol, 1 equiv., pre-swollen in 1,4-dioxane). The reaction mixture was stirred in a sealed vial at 40 °C in a heating mantle for 24 h. The resin was drained and washed with DMF (3 × 10 mL), DCM (3 × 10 mL) and MeOH (3 × 10 mL). After the following oxidation and cleavage, the resulting crude was washed and centrifuged with hexane (3 x 1 mL), then diethyl ether (3 x 3 mL) and the supernatant solutions were decanted to give compound **15** as a purple solid (21 mg, 80%).

^{1}H NMR (500 MHz, DMSO-*d$_6$*) δ 8.65 (d, *J* = 8.5 Hz, 2H), 8.57 (d, *J* = 8.5 Hz, 2H), 8.24 (d, *J* = 8.5 Hz, 2H), 7.77 – 7.68 (m, 3H). **^{13}C NMR** (126 MHz, DMSO-*d$_6$*) δ 166.7, 163.4, 163.0, 135.6, 132.8, 131.7, 130.3, 130.2, 129.5, 127.7, 127.7. **HRMS** (ESI) for $C_{15}H_9N_4O_2$ $[M-H]^-$ *calcd*. 277.0731; *found* 277.0720.

6-(1,2,4,5-Tetrazin-3-yl)nicotinic acid (16)

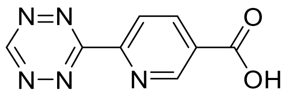

A mixture of DCM (2 mL, 0.05 M), 3-mercaptopropionic acid (25.3 μL,0.29 mmol, 3 equiv.), and hydrazine hydrate (1 mL, 0.1 M) was added to the nitrile functionalized trityl-linker resin (100 mg, 95 μmol, 1 equiv., pre-swollen in DCM). The reaction mixture was stirred in a sealed vial at 40 °C in a heating mantle for 24 h. The resin was drained and washed with DMF (3 × 10 mL), DCM (3 × 10 mL) and MeOH (3 × 10 mL). After oxidation and cleavage, the crude product was purified by column chromatography (Acetone/MeOH 9:1, *v/v*) to give compound **16** as a pink solid (16 mg, 83%). Analytical data in agreement with literature.[2]

^{1}H NMR (500 MHz, DMSO-d_6) δ 10.75 (s, 1H), 9.35 (s, 1H), 8.67 (d, *J* = 8.0 Hz, 1H), 8.58 (d, *J* = 8.0 Hz, 1H). **^{13}C NMR** (126 MHz, DMSO-d_6) δ 165.7, 164.9, 158.5, 153.2, 150.9, 138.7, 128.7, 124.1. **HRMS** (ESI) for $C_8H_6N_5O_2$ [M+H]$^+$ *calcd*. 204.0516; *found* 204.0516.

6-(6-Methyl-1,2,4,5-tetrazin-3-yl)nicotinic acid (17)

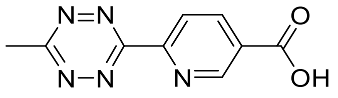

A mixture of acetonitrile (2 mL, 0.05 M), 3-mercaptopropionic acid (25.3 μL, 0.29 mmol, 3 equiv.) and hydrazine hydrate (1 mL, 0.1 M) were added to the nitrile functionalized trityl-linker resin (100 mg, 95 μmol, 1 equiv., pre-swollen in 1,4-dioxane). The reaction mixture was stirred in a sealed vial at 40 °C in a heating mantle for 24 h. The resin was drained and washed with DMF (3 × 10 mL), DCM (3 × 10 mL) and MeOH (3 × 10 mL). After oxidation and cleavage, the crude product was then purified by preparative reverse phase HPLC and the desired fractions were pooled and lyophilized to afford compound **17** as a purple-red solid (18 mg, 85%). Analytical data in agreement with literature.[2]

^{1}H NMR (500 MHz, DMSO-d_6) δ 9.31 (s, 1H), 8.59 (d, *J* = 8.0 Hz, 1H), 8.53 (d, *J* = 8.0 Hz, 1H), 3.06 (s, 3H).**^{13}C NMR** (126 MHz, DMSO-d_6) δ 168.1, 166.6, 163.4, 152.8, 151, 138.8, 123.9, 21.5. **HRMS** (ESI) for $C_9H_8N_5O_2$ [M+H]$^+$ *calcd*. 218.0673; *found* 218.0673.

6-(6-phenyl-1,2,4,5-tetrazin-3-yl)nicotinic acid (18)

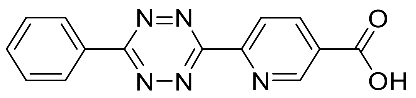

A mixture of benzonitrile (2 mL, 0.05 M), 3-mercaptopropionic acid (25.3 μL, 0.29 mmol, 3 equiv.), and hydrazine hydrate (1 mL, 0.1 M) was added to the nitrile functionalized trityl-linker resin (100 mg, 95 μmol, 1 equiv., pre-swollen in 1,4-dioxane). The reaction mixture was stirred in a sealed vial at 40 °C in a heating mantle for 24 h. The resin was drained and washed with DMF (3 × 10 mL), DCM (3 × 10 mL) and MeOH (3 × 10 mL). After the following oxidation and cleavage, the resulting crude was washed and centrifuged with DCM (3 x 3 mL), then hexane (3 x 3 mL) and the supernatant solutions were decanted to give compound **18** as a purple solid (20.7 mg, 78%).

^{1}H NMR (500 MHz, DMF-d_7) δ 9.47 (s, 1H), 8.77 (d, *J* = 8.4 Hz, 1H), 8.70 – 8.64 (m, 3H), 7.82 – 7.73 (m, 3H). **^{13}C NMR** (126 MHz, DMF-d_7) δ 167.2, 165.1, 164.7, 153.8, 150.9, 137.9, 134.1, 133.2, 133.1, 130.7, 129.2, 124.7. **HRMS** (ESI) for $C_{14}H_{10}N_5O_2$ $[M+H]^+$ *calcd*. 280.0829; *found* 280.0829.

6-(6-(5-Bromopyridin-3-yl)-1,2,4,5-tetrazin-3-yl)nicotinic acid (19)

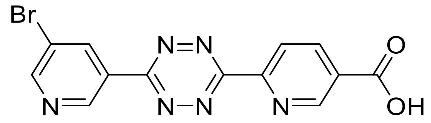

A mixture of 5-bromo-3-cyanopyridine (53 mg, 0.29 mmol, 3 equiv.) in 1,4-dioxane (2 mL, 0.03 M), 3-mercaptopropionic acid (25.3 μL, 0.29 mmol, 3 equiv.), and hydrazine hydrate (1 mL, 0.1 M) was added to the nitrile functionalized trityl linker resin (100 mg, 95 μmol, 1 equiv., pre-swollen in 1,4-dioxane). The reaction mixture was stirred in a sealed vial at 40 °C in a heating mantle for 24 h. The resin was drained and washed with DMF (3 × 10 mL), DCM (3 × 10 mL) and MeOH (3 × 10 mL). After the following oxidation and cleavage, the crude product was purified by column chromatography (DCM/MeOH/AcOH 90:10:1, *v/v/v*) to give compound **19** as a pink solid (29 mg, 85%).

^{1}H NMR (600 MHz, DMSO-d_6) δ 9.62 (d, *J* = 1.9 Hz, 1H), 9.36 (dd, *J* = 2.0, 0.9 Hz, 1H), 9.07 (d, *J* = 2.3 Hz, 1H), 9.00 (t, *J* = 2.1 Hz, 1H), 8.63 (dd, *J* = 8.0, 0.9 Hz, 1H), 8.53 (dd, *J* = 8.0, 2.0 Hz, 1H). **^{13}C NMR** (126 MHz, DMSO-d_6) δ 166.1, 163.4, 161.8, 153.8, 151.4, 150.6, 147.2, 138.0, 137.4, 129.8, 123.8, 120.9. **HRMS** (ESI) for $C_{13}H_7BrN_6O_2$ $[M-H]^-$ *calcd*. 357.9774; *found* 357.9753.

6-(6-(Pyridin-2-yl)-1,2,4,5-tetrazin-3-yl)nicotinic acid (20)

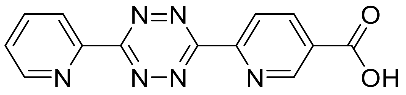

A mixture of 2-cyanopyridine (2 mL, 0.05 M), 3-mercaptopropionic acid (25.3 μL, 0.29 mmol, 3 equiv.), and hydrazine hydrate (1 mL, 0.1 M) was added to the nitrile functionalized trityl linker resin (100 mg, 95 μmol, 1 equiv., pre-swollen in 1,4-dioxane). The reaction mixture was stirred in a sealed vial at 40 °C in a heating mantle for 24 h. The resin was drained and washed with DMF (3 × 10 mL), DCM (3 × 10 mL) and MeOH (3 × 10 mL). After the following oxidation and cleavage, the crude product was purified by column chromatography (DCM/MeOH/AcOH 95:5:1, *v/v/v*) to give compound **20** as a pink solid (24 mg, 90%). Analytical data in agreement with literature.[3]

^{1}H NMR (500 MHz, DMF-d_7) δ 9.46 (dd, *J* = 2.1, 0.9 Hz, 1H), 9.00 (dt, *J* = 4.7, 1.8, 0.9 Hz, 1H), 8.82 (d, *J* = 8.0 Hz, 1H), 8.74 – 8.67 (m, 2H), 8.24 (td, *J* = 7.7, 1.8 Hz, 1H), 7.82 – 7.76 (ddd, *J* = 7.7, 4.7, 0.9 Hz, 1H). **^{13}C NMR** (126 MHz, DMF-d_7) δ 173.8, 164.9, 164.8, 152.4, 151.9, 151.8, 146.7, 139.6, 138.8, 137.8, 127.8, 125.7, 125.2. **HRMS** (ESI) for $C_{13}H_8N_6O_2$ $[M-H]^-$ *calcd*. 279.0636; *found* 279.0618.

6,6'-(1,2,4,5-Tetrazine-3,6-diyl)dinicotinic acid (21)

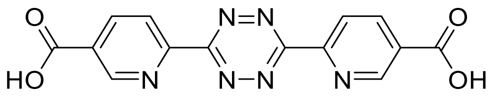

A mixture of 6-cyanonicotinic acid (43 mg, 0.29 mmol, 3 equiv.) in 1,4-dioxane (2 mL, 0.03 M), 3-mercaptopropionic acid (25.3 μL, 0.29 mmol, 3 equiv.), and hydrazine hydrate (1 mL, 0.1 M) was added to the nitrile functionalized trityl linker resin (100 mg, 95 μmol, 1 equiv., pre-swollen in 1,4-dioxane). The reaction mixture was stirred in a sealed vial at 40 °C in a heating mantle for 24 h. The resin was drained and washed with DMF (3 × 10 mL), DCM (3 × 10 mL) and MeOH (3 × 10 mL). After the following oxidation and cleavage, the resulting crude was washed and centrifuged with DMSO (3 × 1 mL), acetone (3 × 3 mL) then DCM (3 × 3 mL) and the supernatant solutions were decanted to give compound **21** as a pink solid (29 mg, 94%).

^{1}H NMR (600 MHz, Acetic acid-*d4*) δ 9.34 (dd, *J* = 2.1, 0.9 Hz, 2H), 8.59 (dd, *J* = 8.0, 2.1 Hz, 2H), 8.00 (dd, *J* = 8.0, 0.9 Hz, 2H). **^{13}C NMR** (151 MHz, Acetic acid-*d4*) δ 167.0, 151.6, 139.1, 136.6, 128.6, 128.6, 116.1. **HRMS** (ESI) for $C_{14}H_8N_6O_4$ $[M-H]^-$ *calcd*. 323.0534; *found* 323.0514.

Scaling–up:

(i). A mixture of DCM (4 mL, 0.15 M), 3-mercaptopropionic acid (156.9 μL, 3 mmol, 3 equiv.), and hydrazine hydrate (1 mL, 0.3 M) was added to the benzonitrile functionalized Rink resin (1 g, 0.6 mmol, 1 equiv., pre-swollen in DCM). The reaction mixture was stirred in a sealed vial at 40 °C in a heating mantle for 24 h. The resin was drained and washed with DMF (3 × 10 mL), DCM (3 × 10 mL) and MeOH (3 × 10 mL). After oxidation and cleavage, the crude product was purified by column chromatography (Petroleum ether/EtOAc/AcOH 50:50:1, *v/v/v*) to give compound **4** as a pink solid (106 mg, 88%).

(ii). A mixture of DCM (4 mL, 0.25 M), 3-mercaptopropionic acid (261.4 μL, 3 mmol, 3 equiv.), and hydrazine hydrate (1 mL, 0.5 M) was added to the benzonitrile functionalized Rink resin (1 g, 1 mmol, 1 equiv., pre-swollen in DCM). The reaction mixture was stirred in a sealed vial

at 40 °C in a heating mantle for 24 h. The resin was drained and washed with DMF (3 × 10 mL), DCM (3 × 10 mL) and MeOH (3 × 10 mL). After oxidation and cleavage, the crude product was purified by column chromatography (Petroleum ether/EtOAc/AcOH 50:50:1, *v/v/v*) to give compound **4** as a pink solid (180 mg, 90%).

Table S1: Compound 4 scaling studies

Entry	CM-Resin Loading	CM-Resin amount	Theoretical Yield	Experimental Yield
**1**	0.6 mmol/g	0.1 g	12 mg	10 mg (86%)
**2**	0.6 mml/g	1 g	121 mg	106 mg (88%)
**3**	1 mmol/g	1 g	201 mg	180 mg (90%)

4. ^{1}H and ^{13}C NMR Spectra

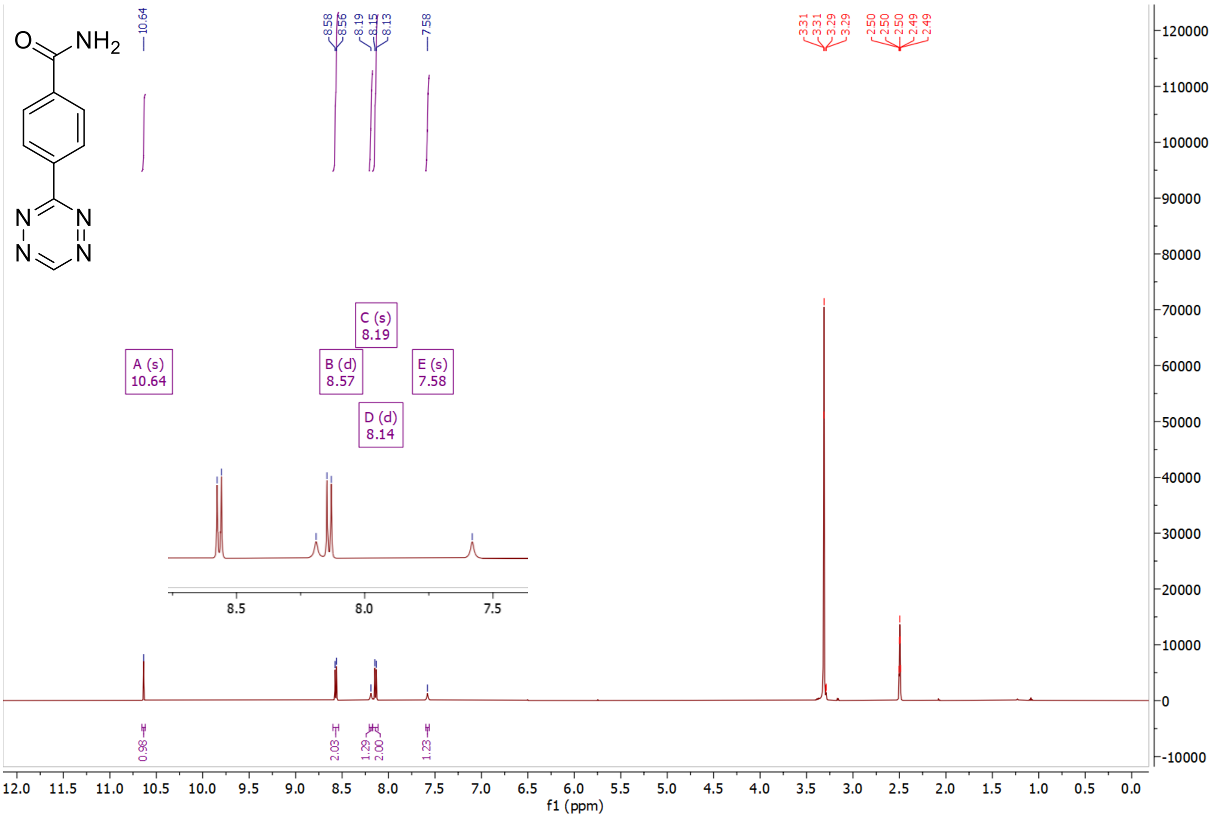

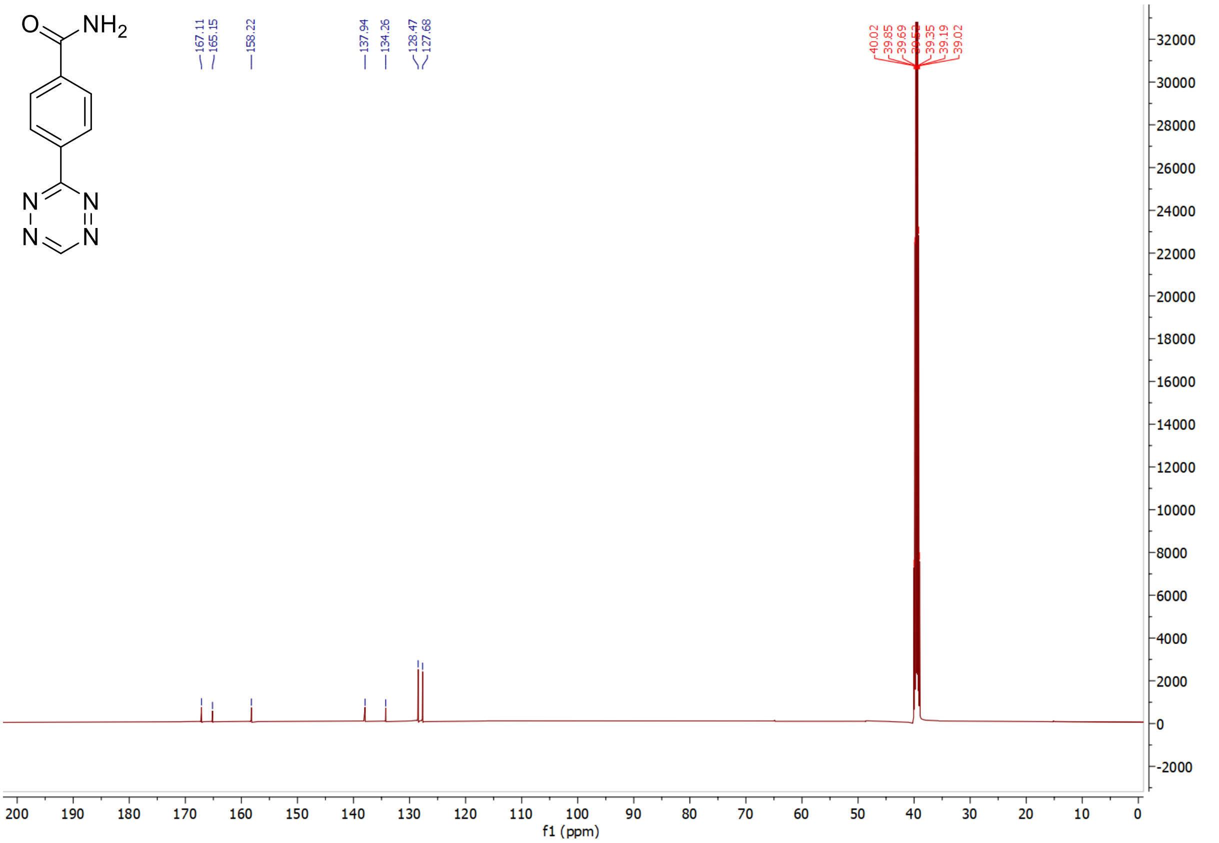

Figure S2. ^{1}H and ^{13}C NMR spectra of compound **4** recorded in DMSO-d_6 at 500 MHz and 126 MHz, respectively.

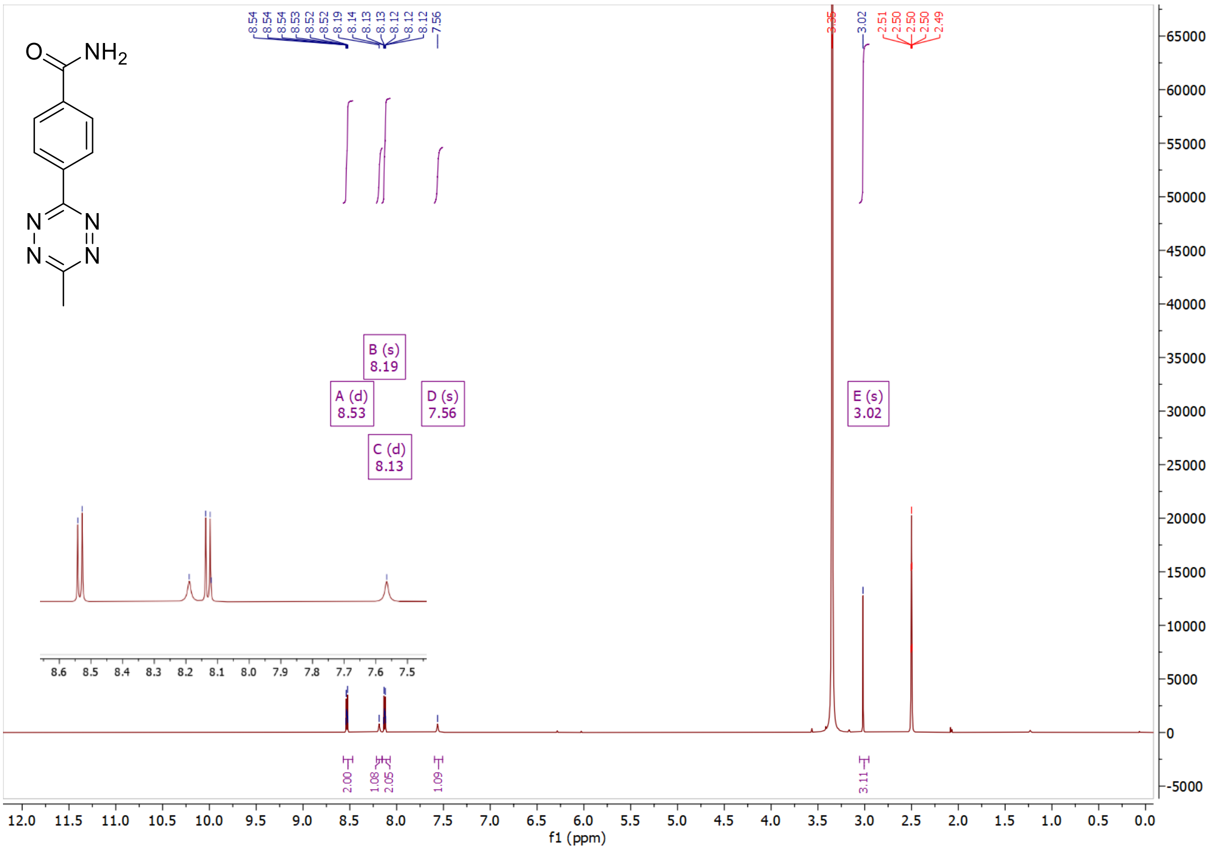

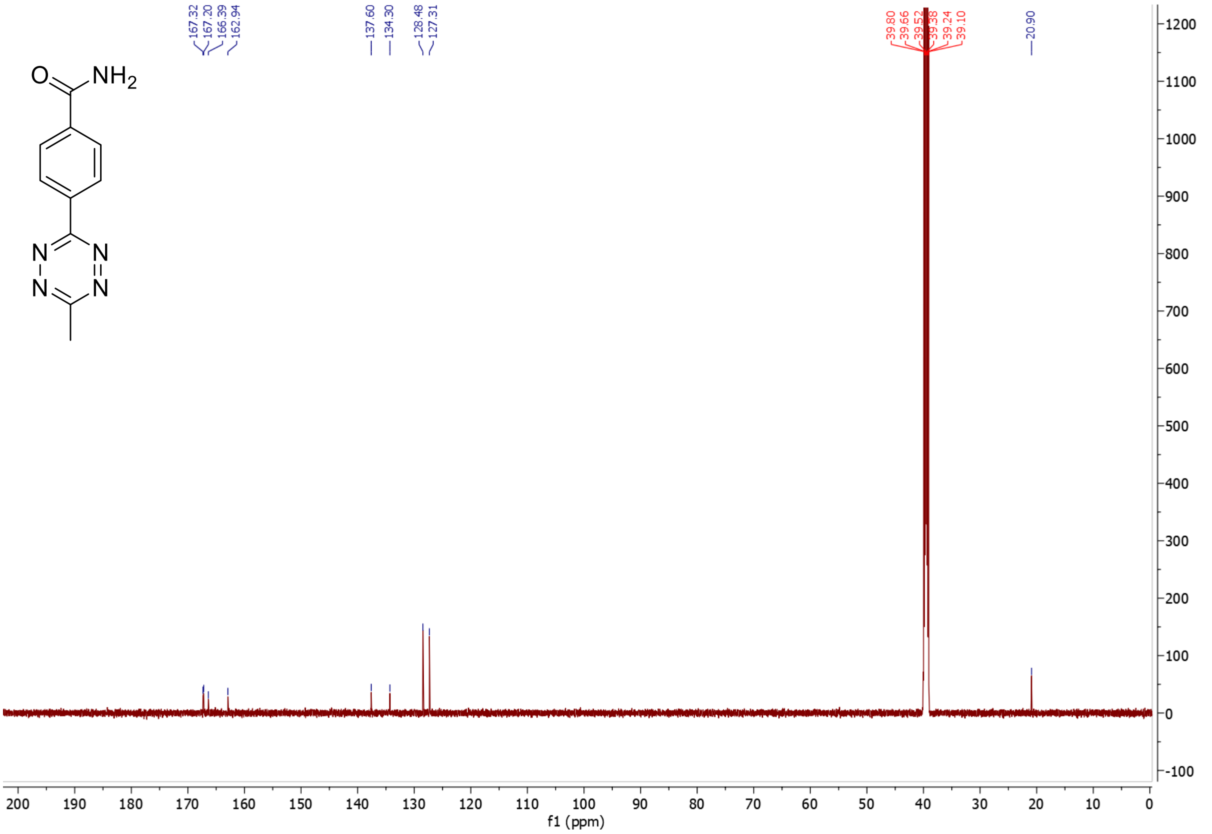

Figure S3. ^{1}H and ^{13}C NMR spectra of compound **5** recorded in DMSO-d_6 at 600 MHz and 151 MHz, respectively.

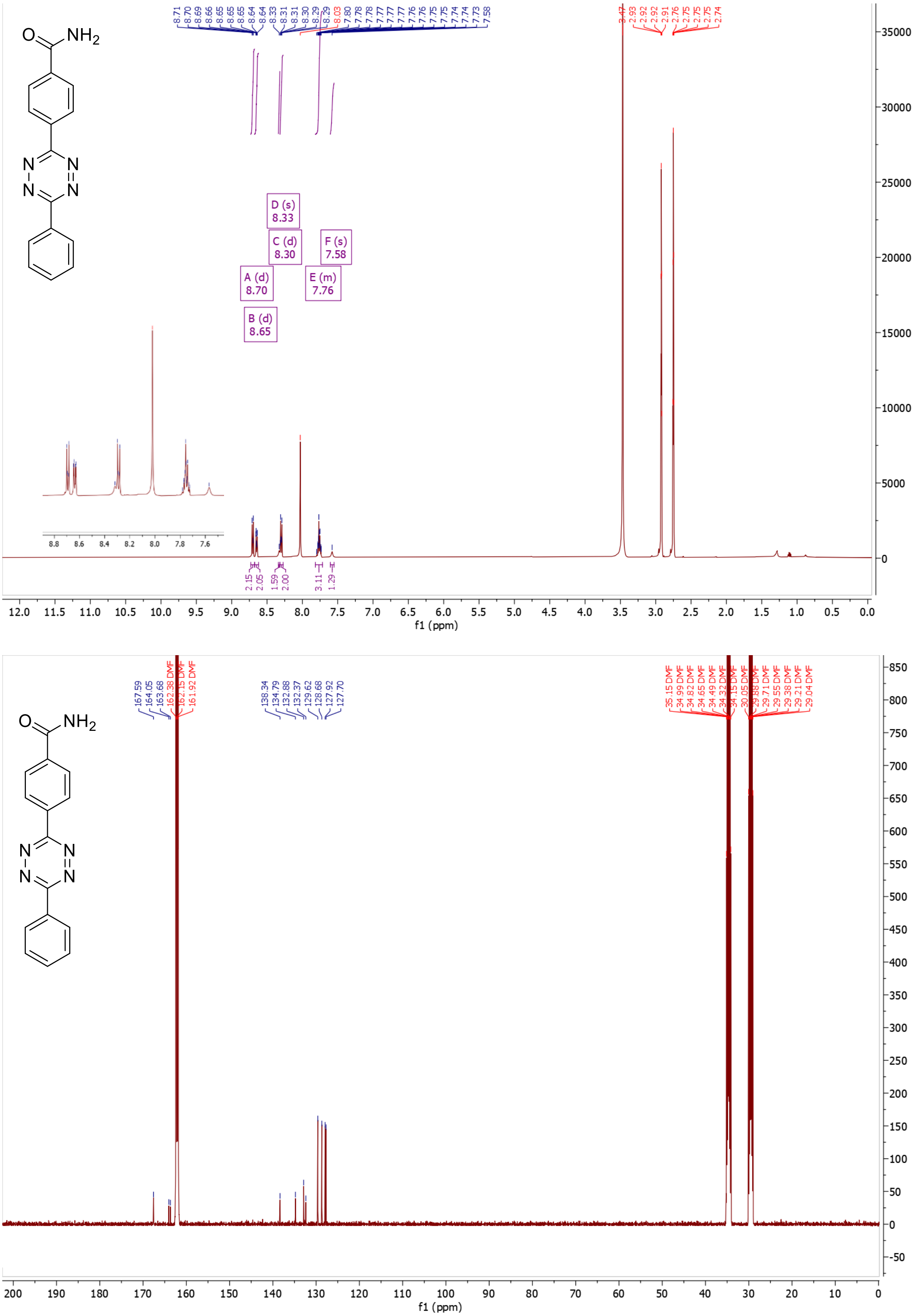

Figure S4. ^{1}H and ^{13}C NMR spectra of compound **7** recorded in DMSO-d_6 at 600 MHz and 151 MHz, respectively.

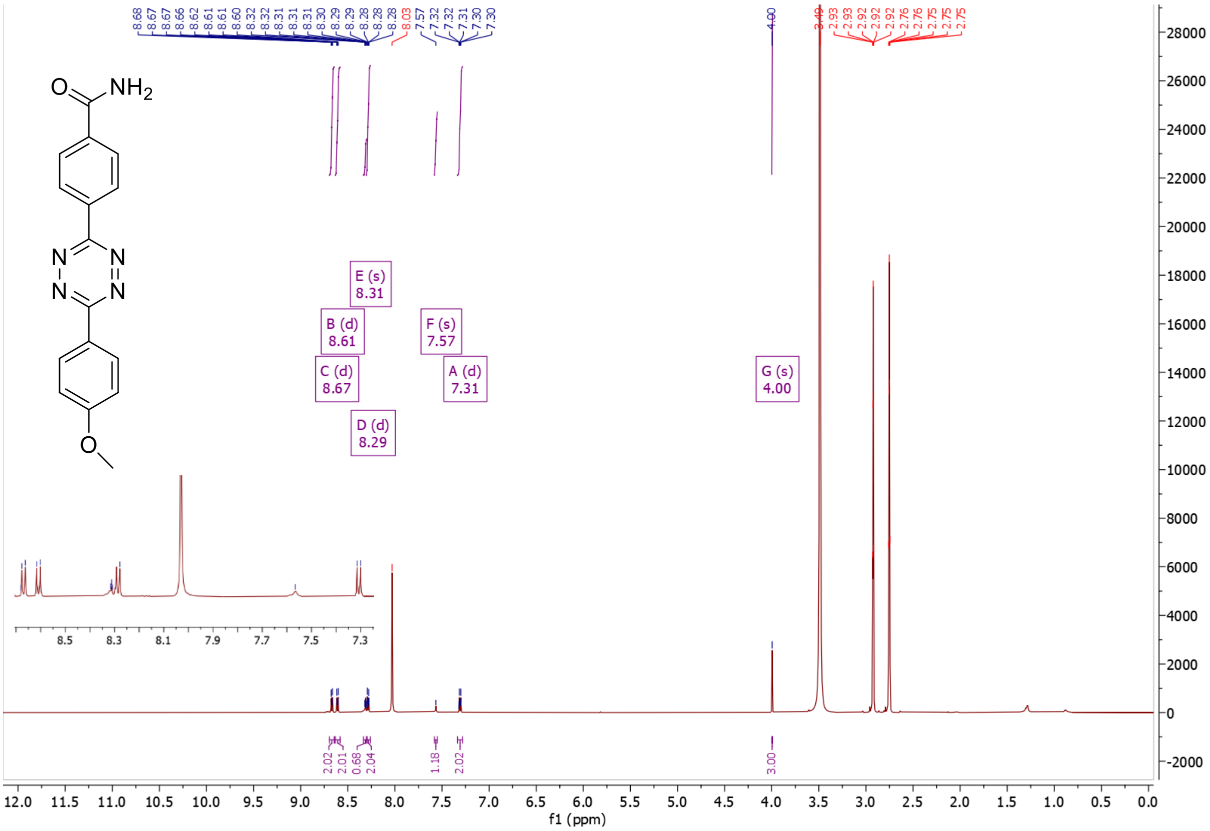

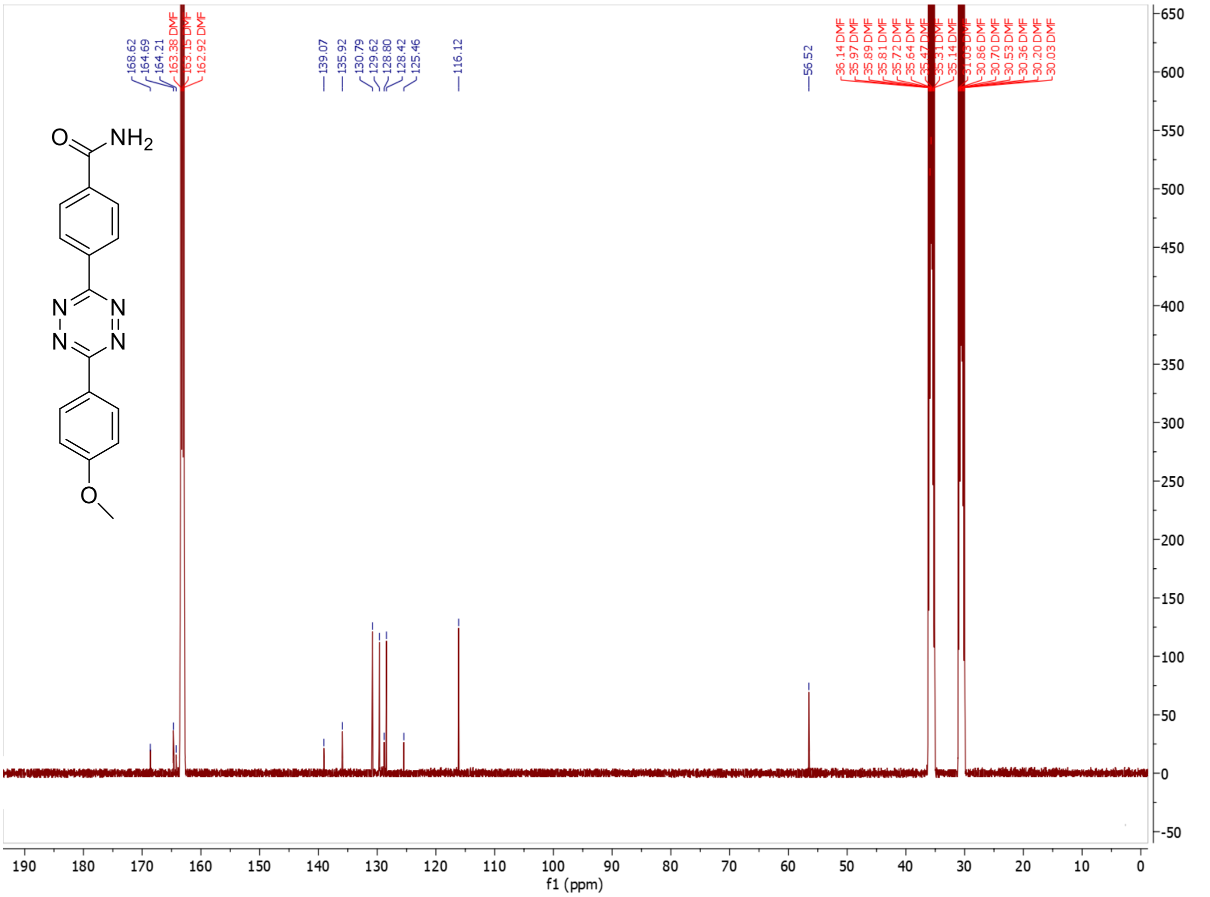

Figure S5. ^{1}H and ^{13}C NMR spectra of compound **8** recorded in DMF-d_7 at 600 MHz and 126 MHz, respectively.

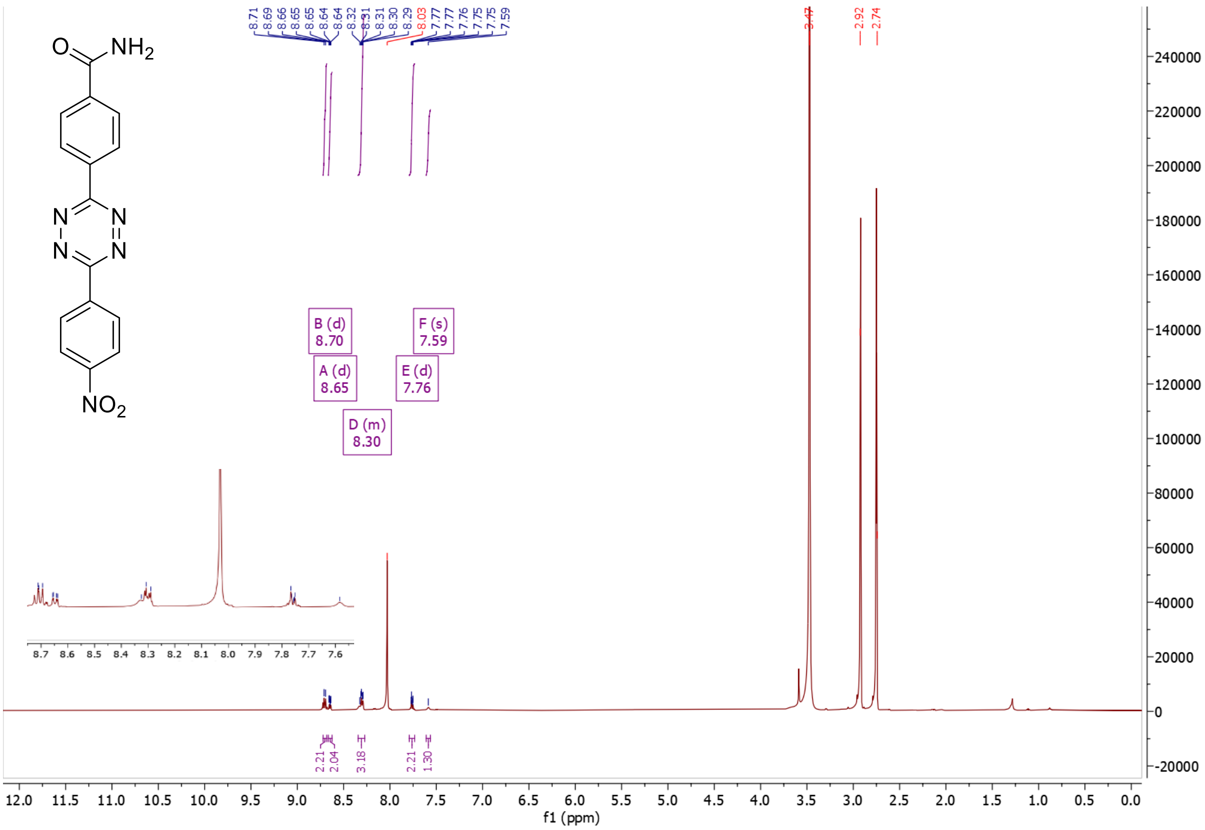

Figure S6. ^{1}H spectra of compound **9** recorded in DMF-d_7 at 500 MHz.

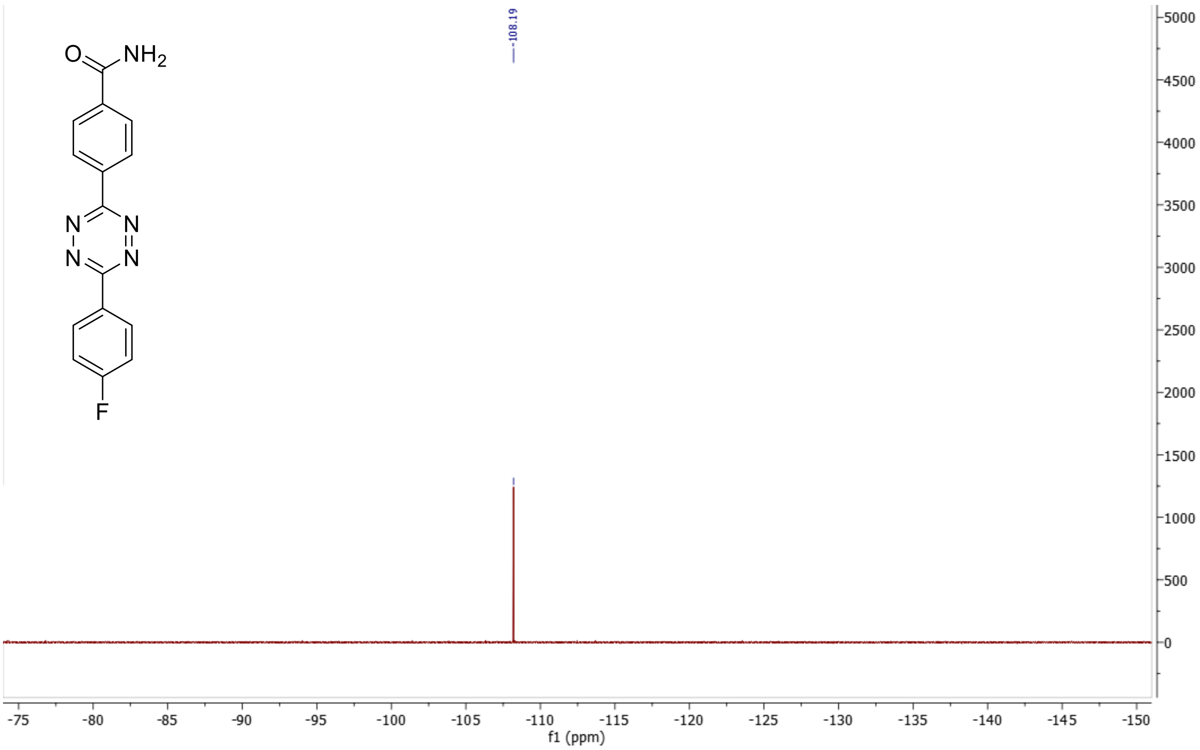

Figure S7. ^{19}F spectra of compound **10** recorded in DMF-d_7 at 400 MHz.

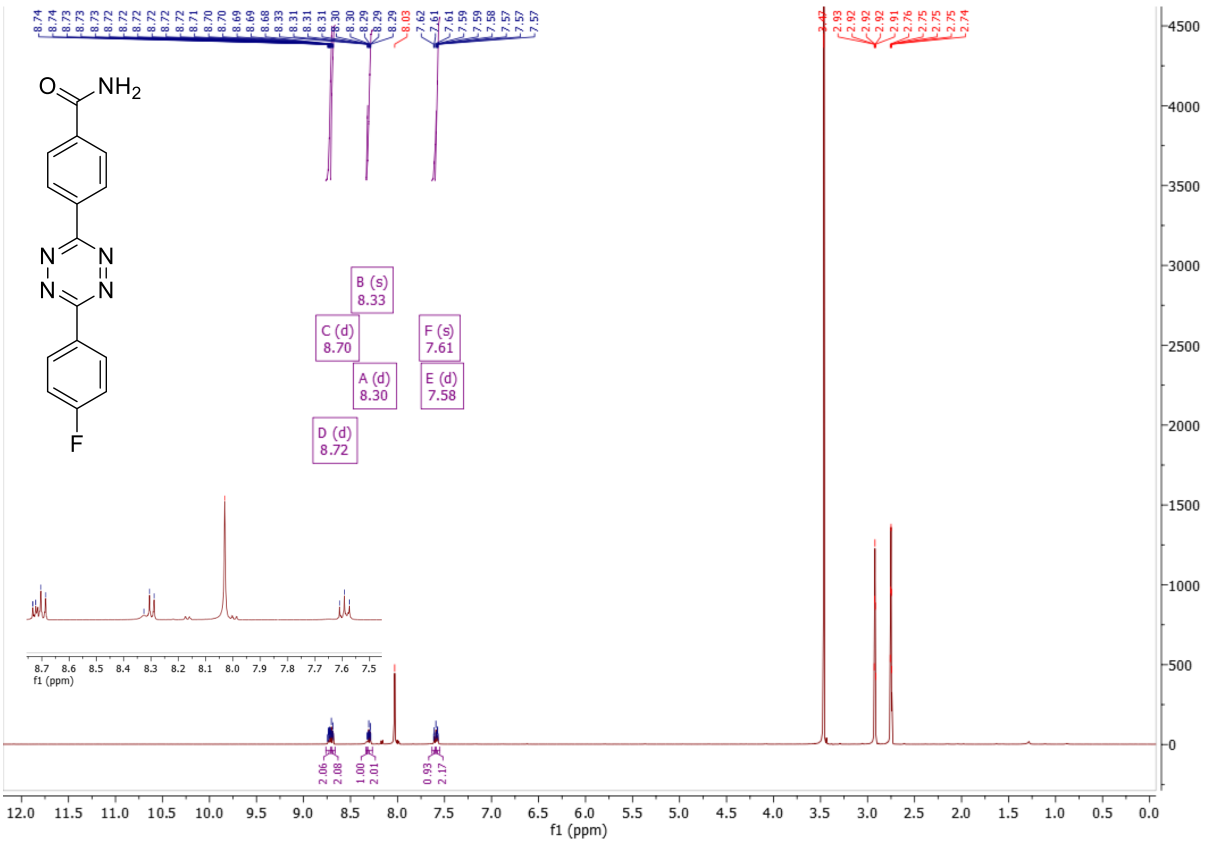

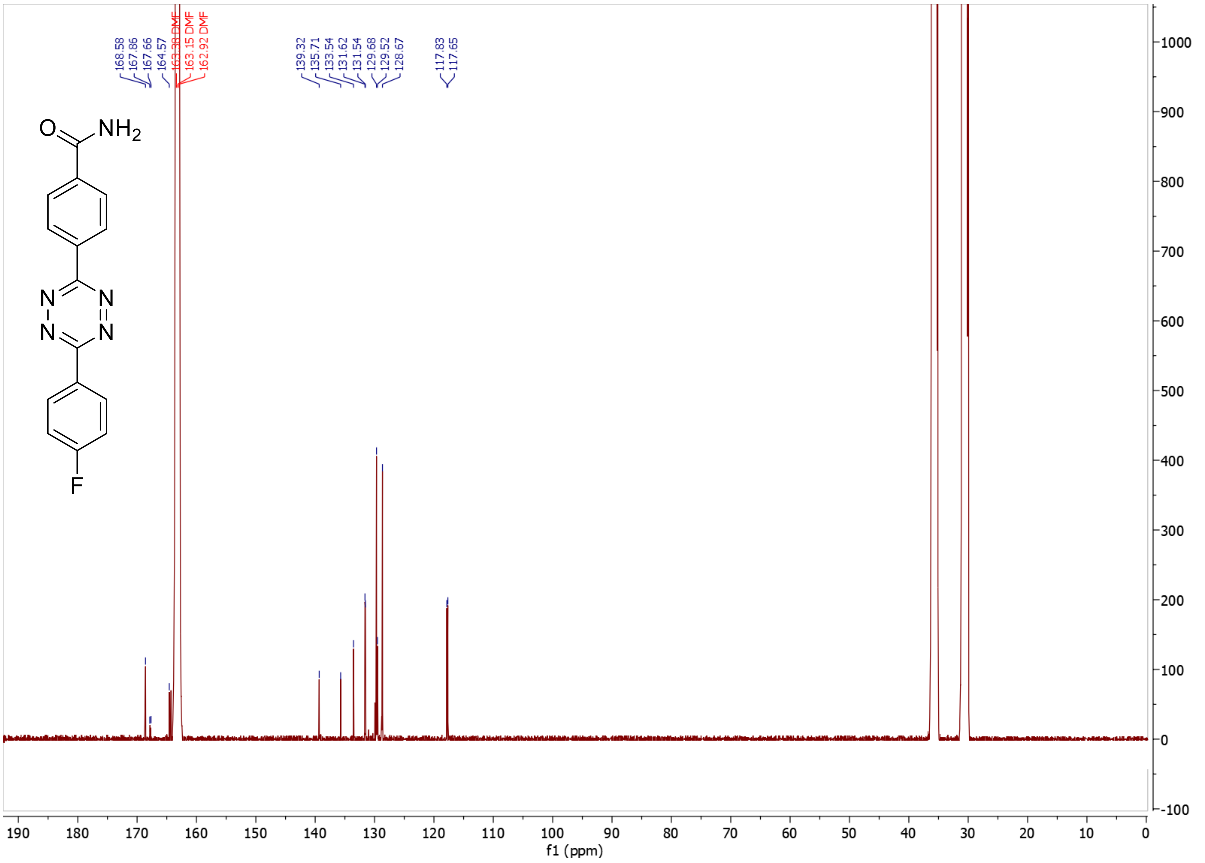

Figure S8. ^{1}H and ^{13}C NMR spectra of compound **10** recorded in DMF-d_7 at 500 MHz and 126 MHz, respectively.

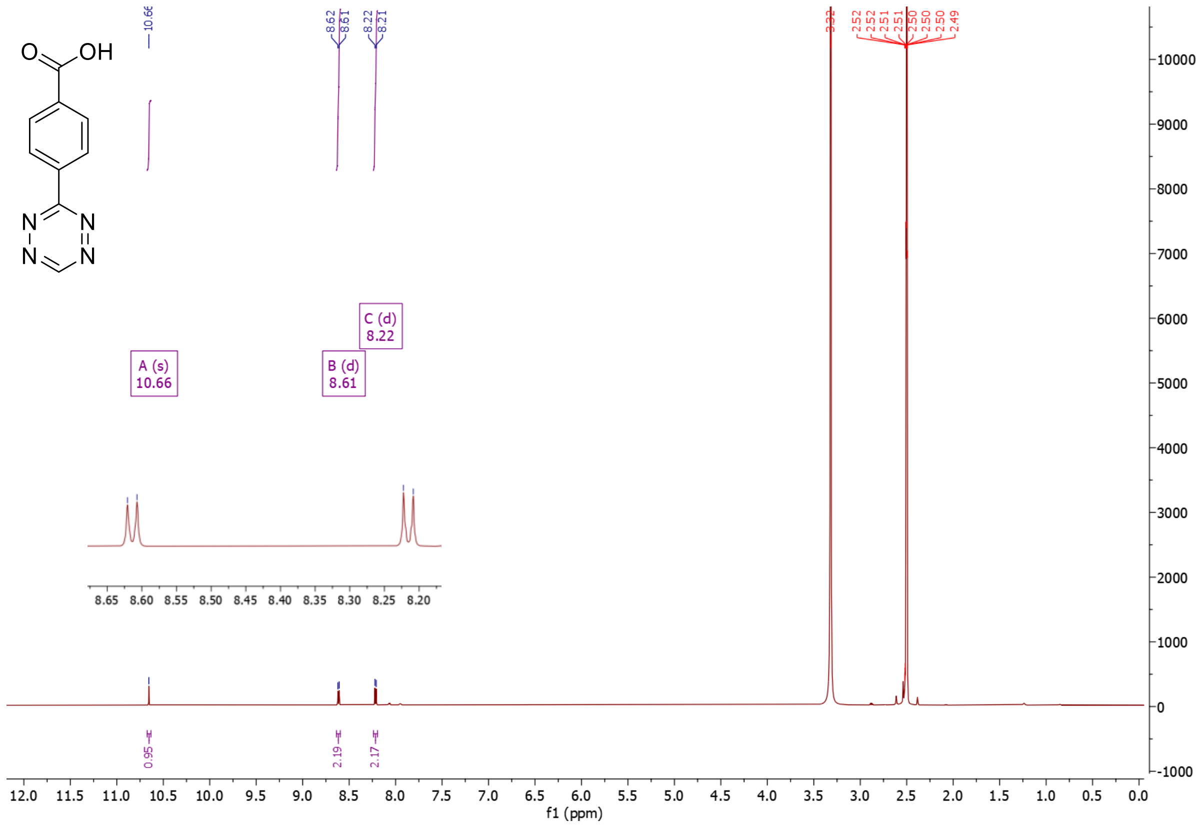

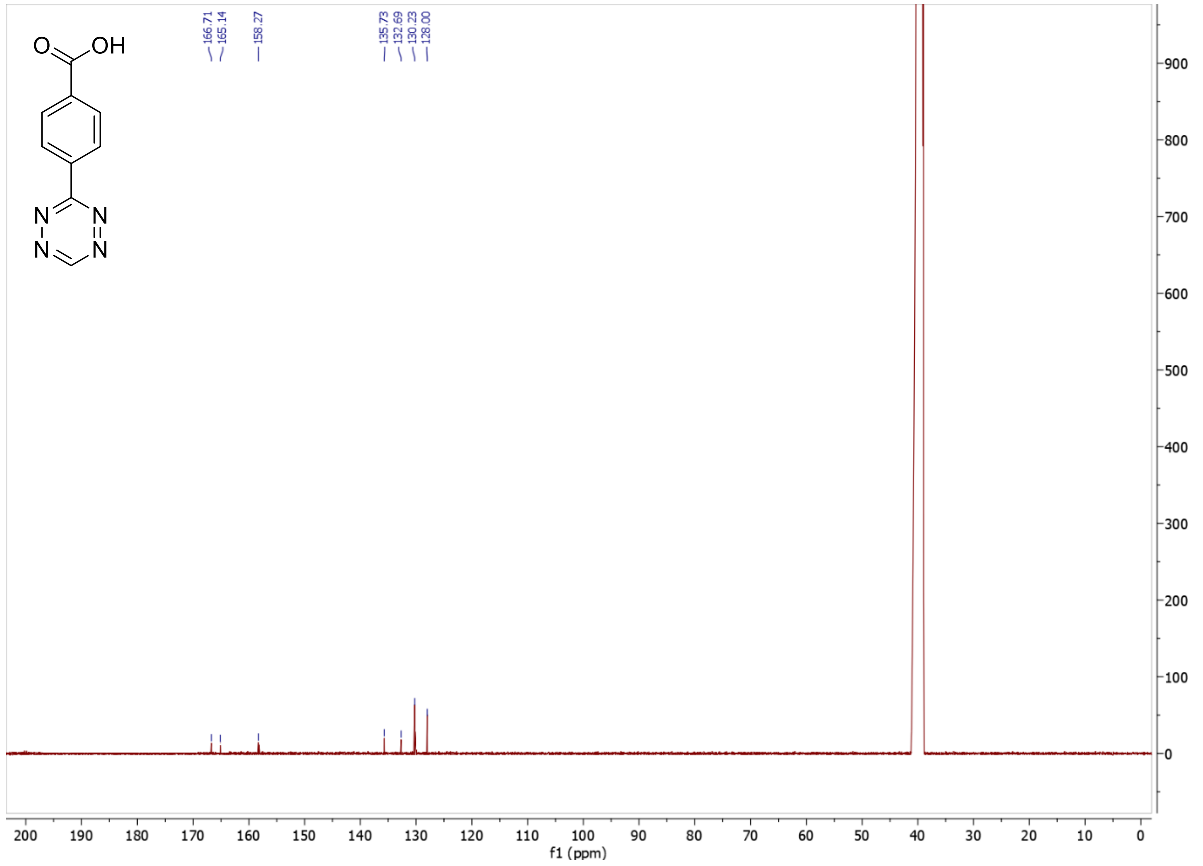

Figure S9. ^{1}H and ^{13}C NMR spectra of compound **13** recorded in DMSO-d_6 at 600 MHz and 126 MHz, respectively.

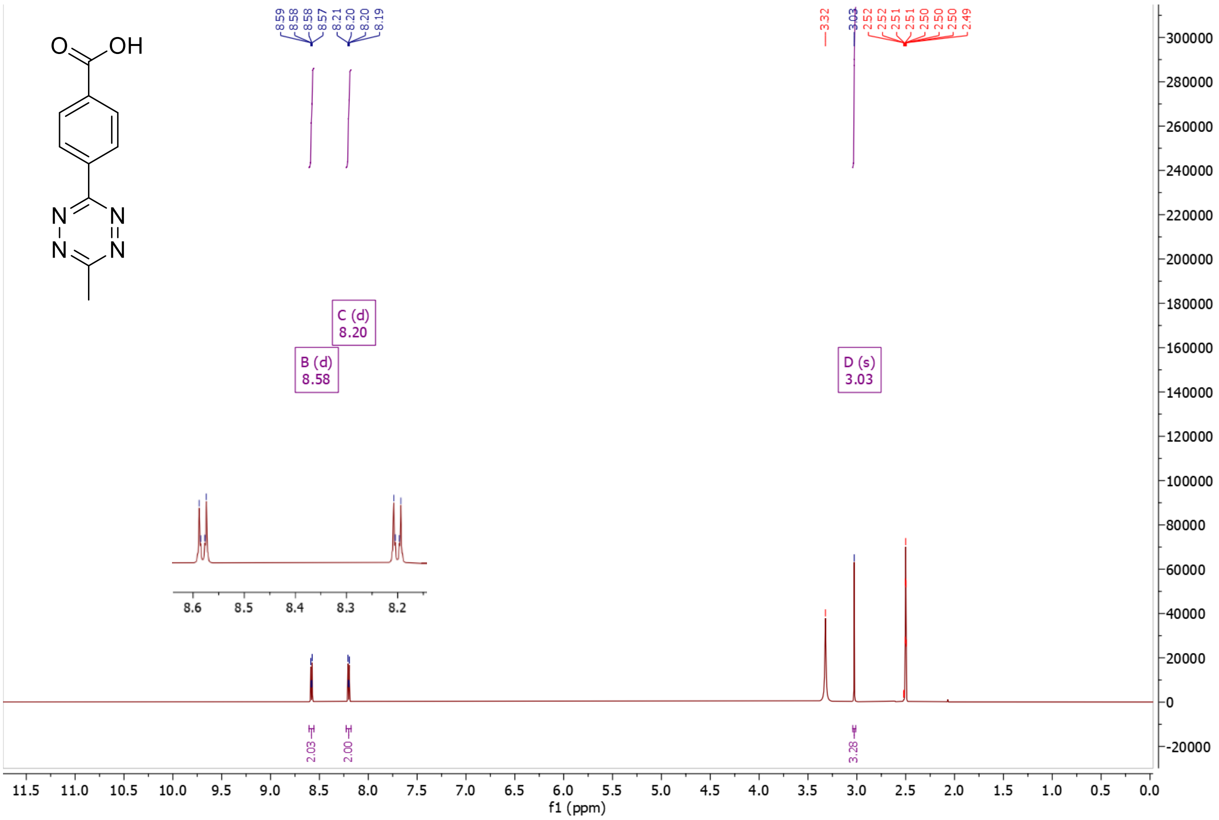

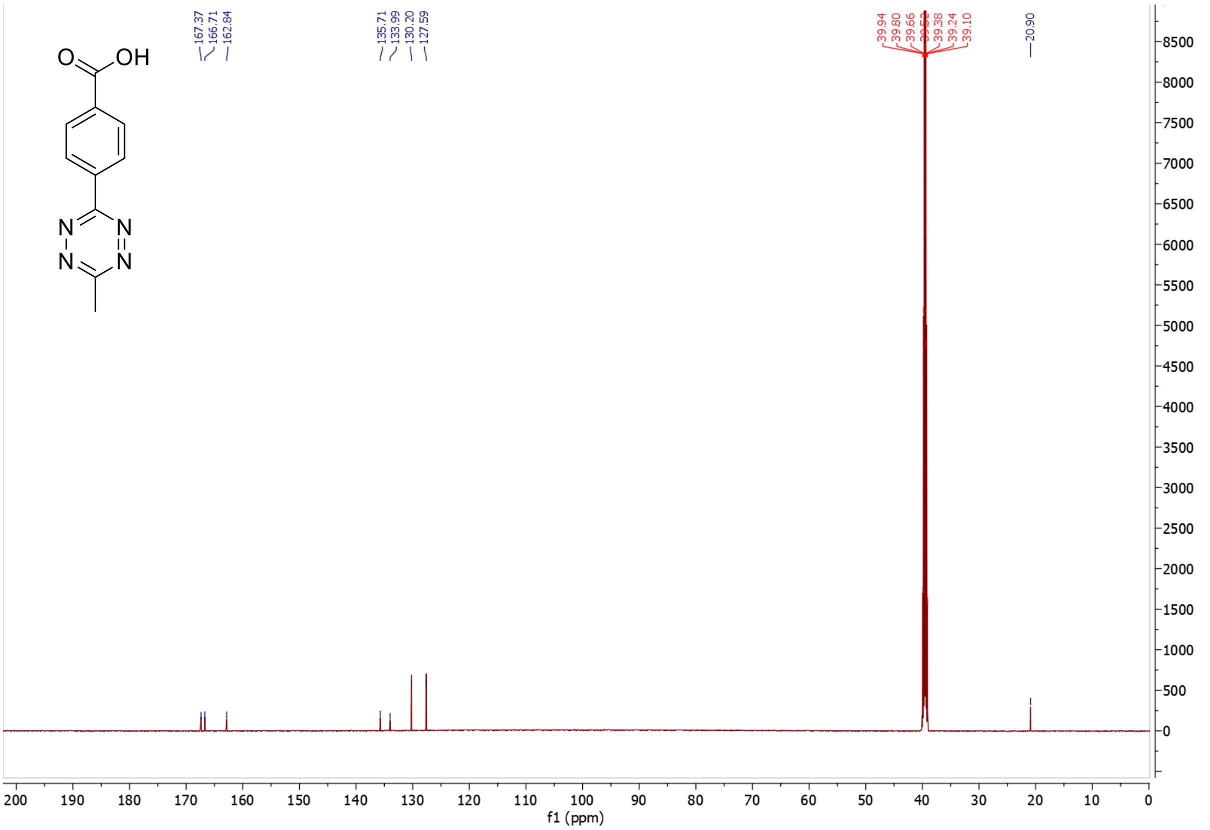

Figure S10. ^{1}H and ^{13}C NMR spectra of compound **14** recorded in DMSO-d_6 at 600 MHz and 151 MHz, respectively.

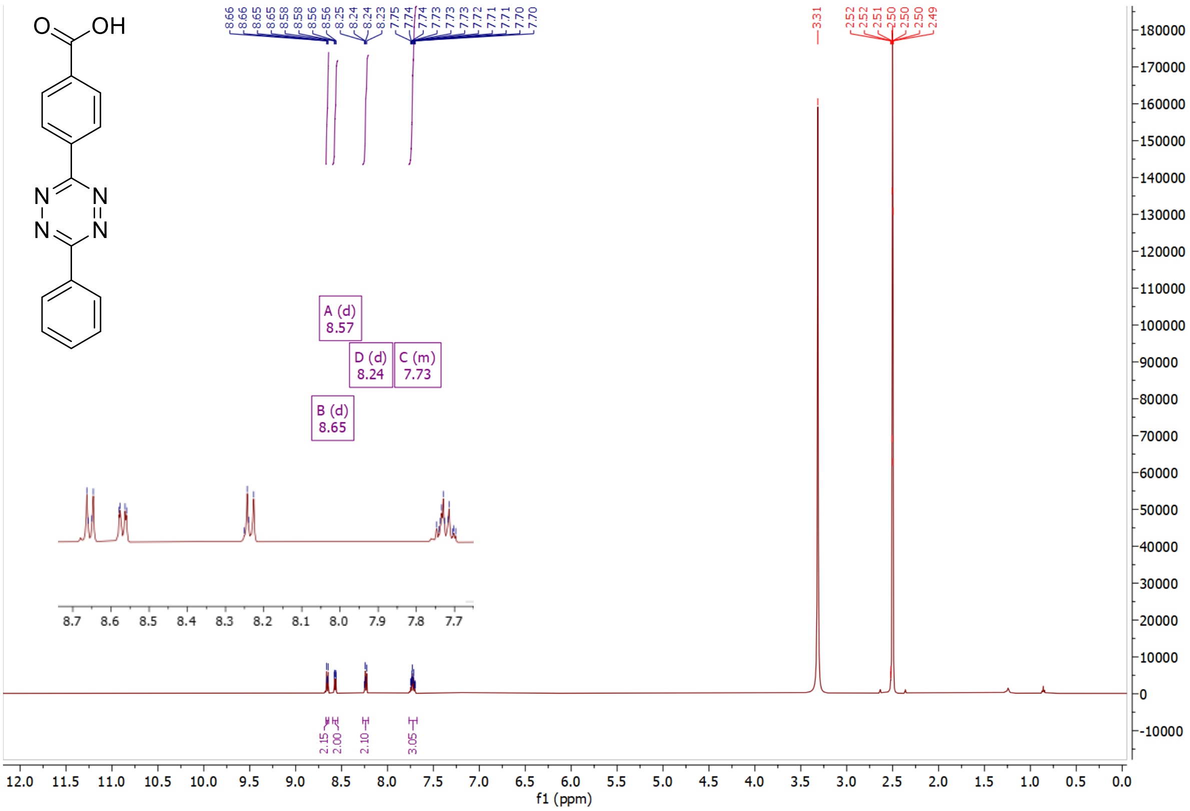

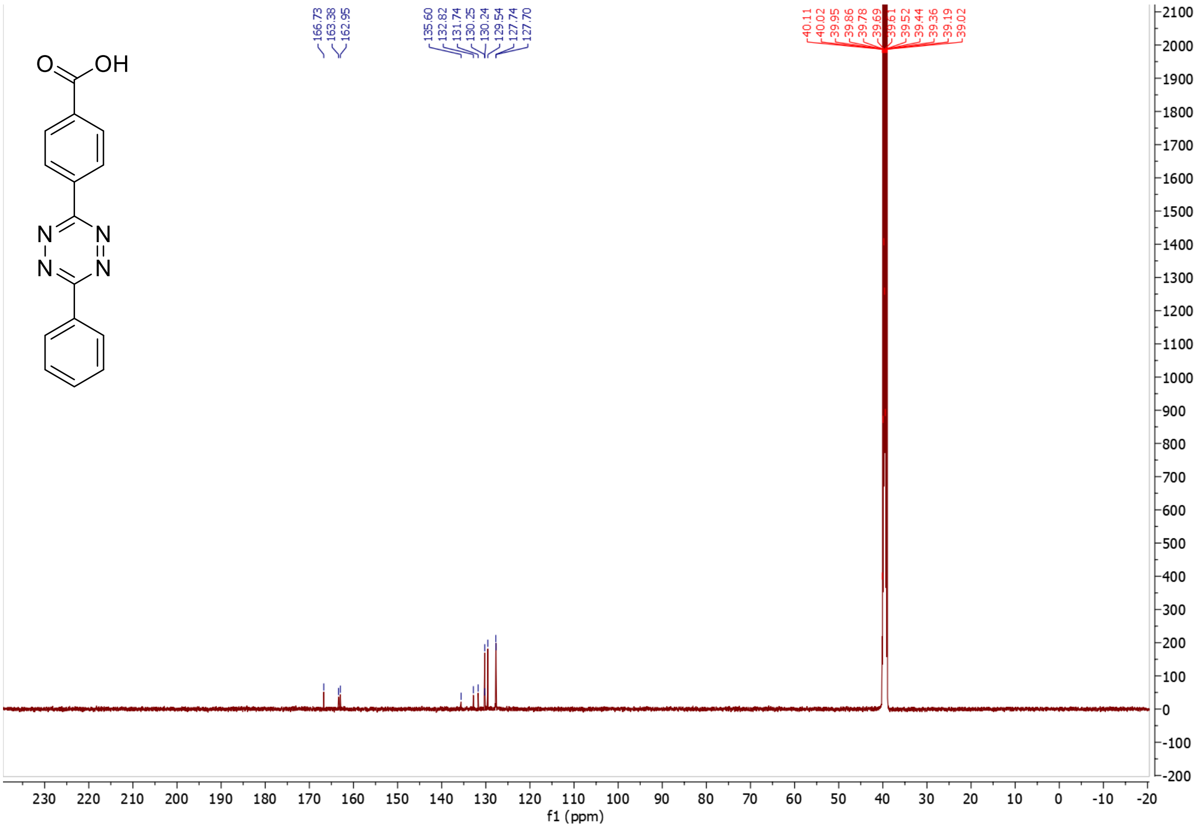

Figure S11. ^{1}H and ^{13}C NMR spectra of compound **15** recorded in DMSO-d_6 at 500 MHz and 126 MHz, respectively.

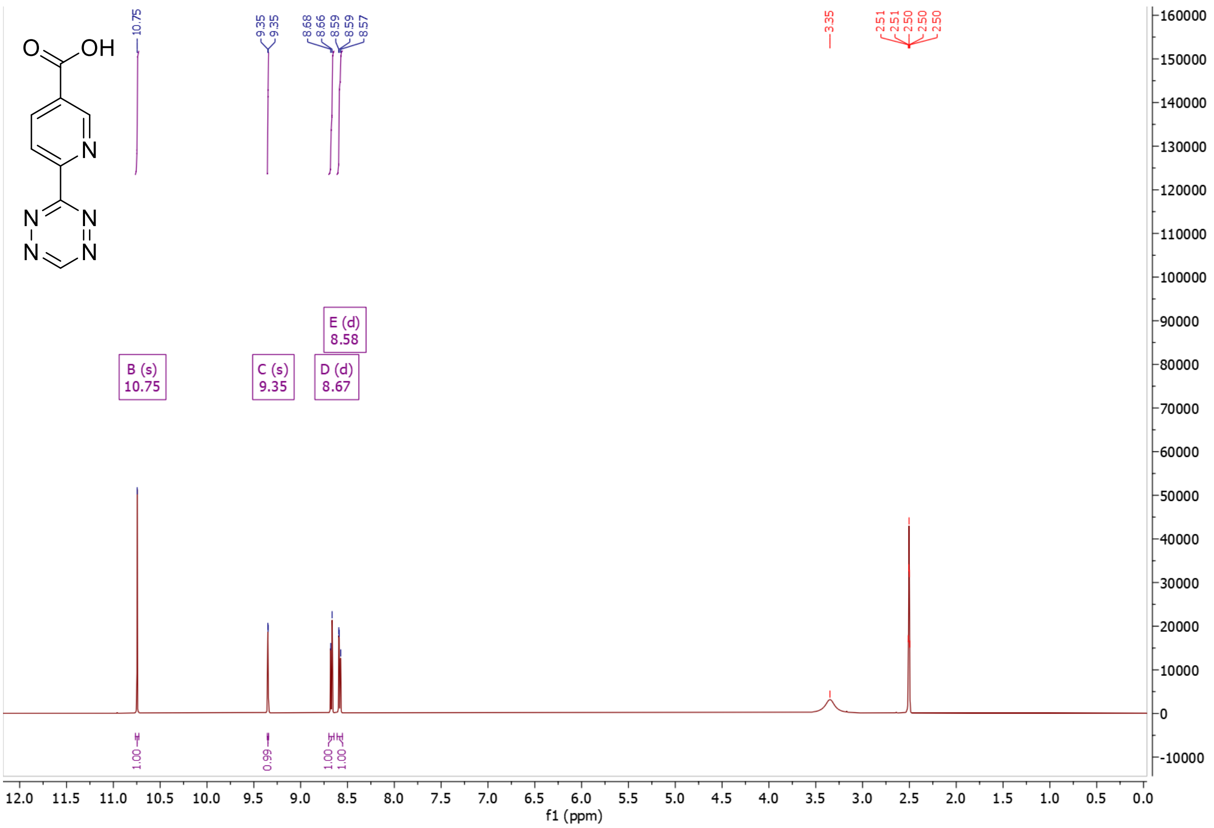

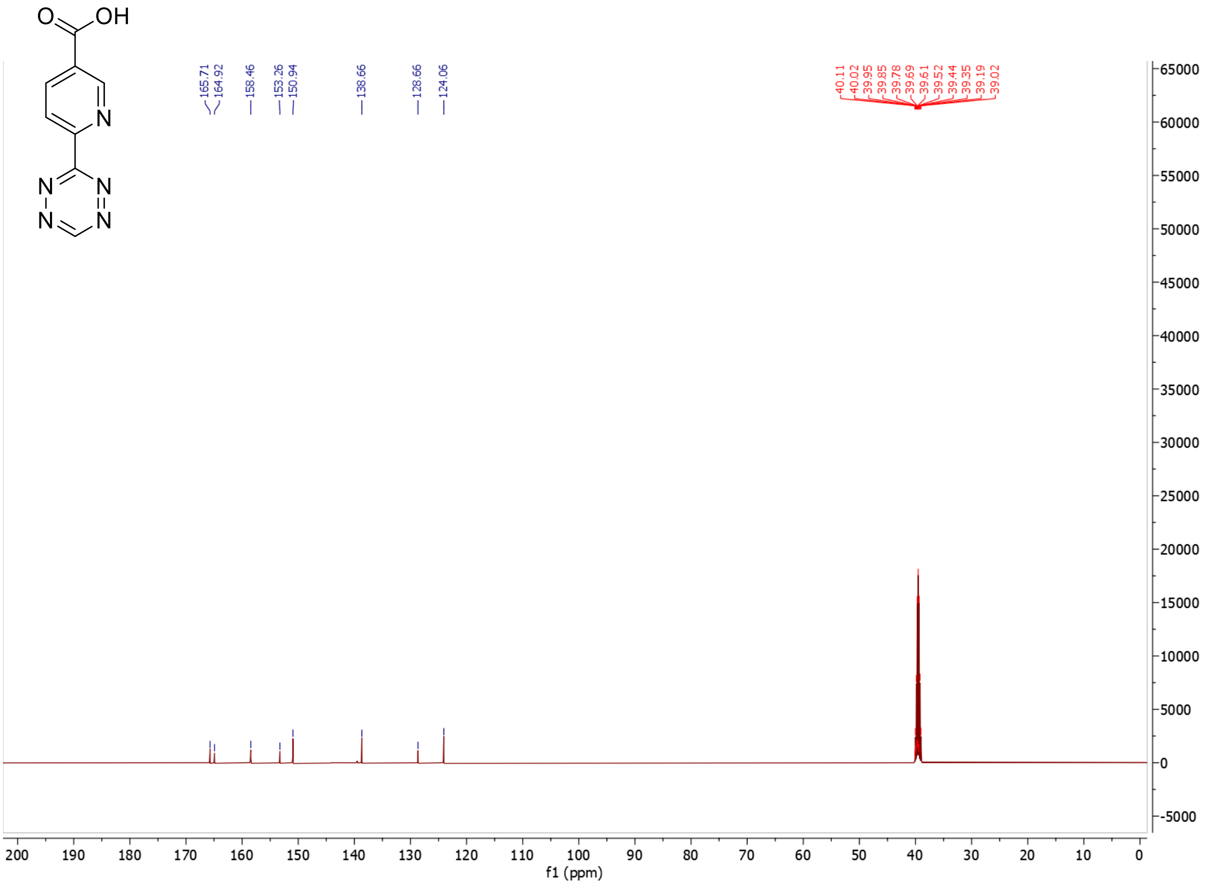

Figure S12. ^{1}H and ^{13}C NMR spectra of compound **16** recorded in DMSO-d_6 at 500 MHz and 126 MHz, respectively.

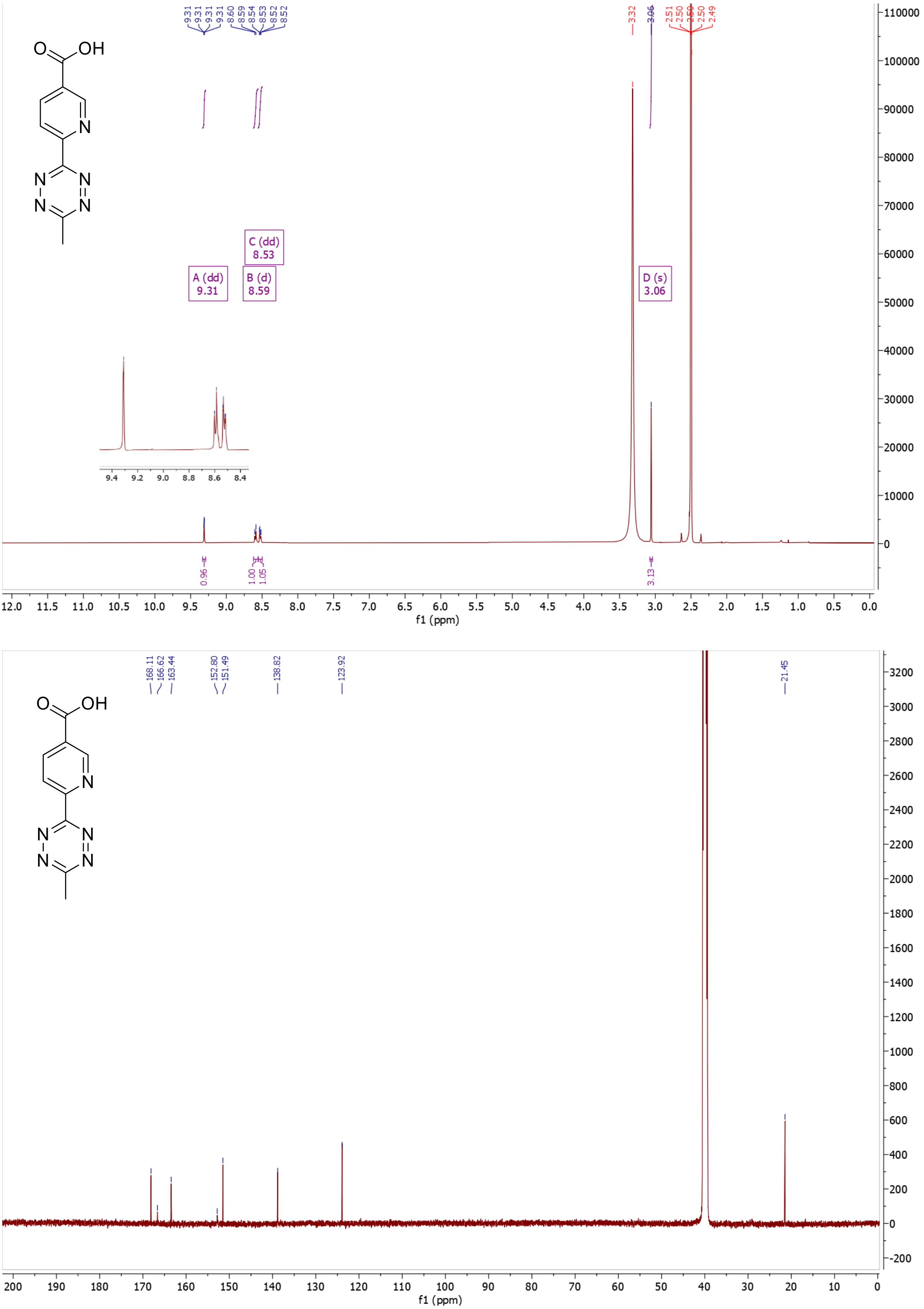

Figure S13. ^{1}H and ^{13}C NMR spectra of compound **17** recorded in DMSO-d_6 at 500 MHz and 126 MHz, respectively

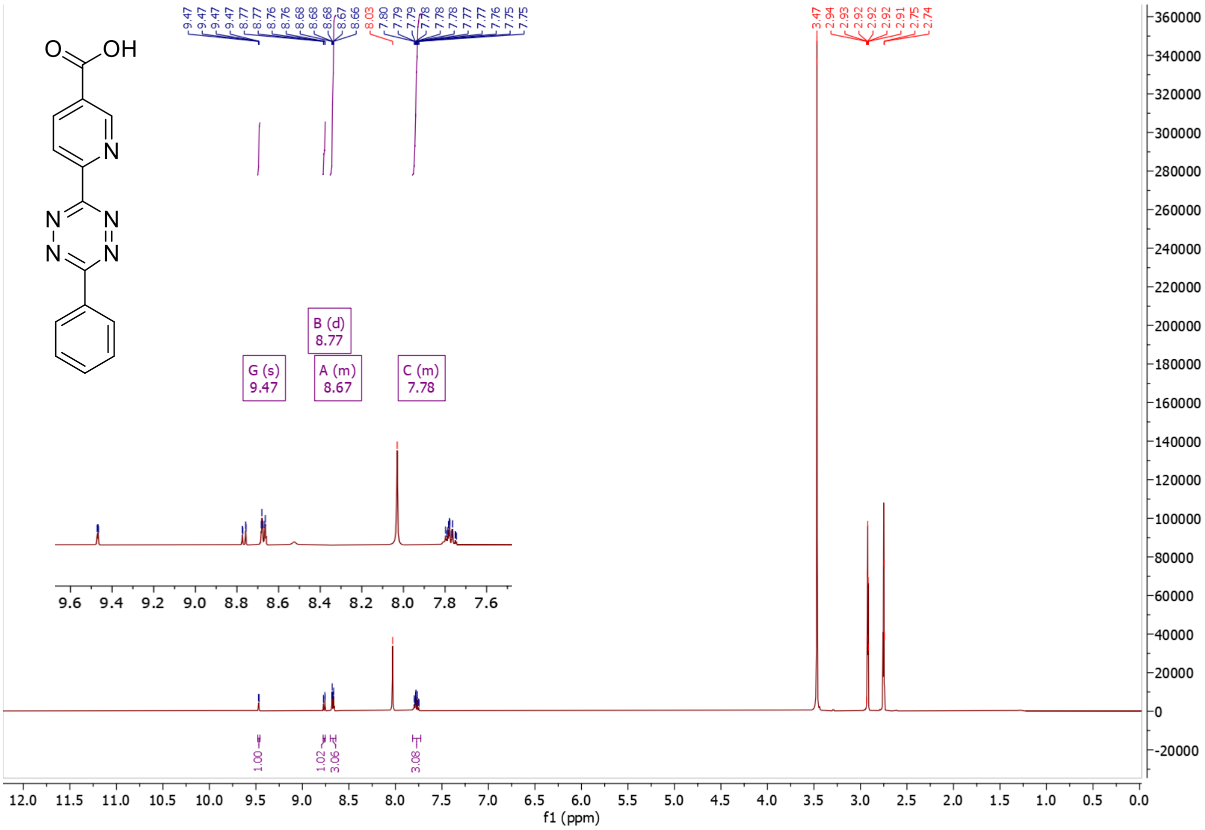

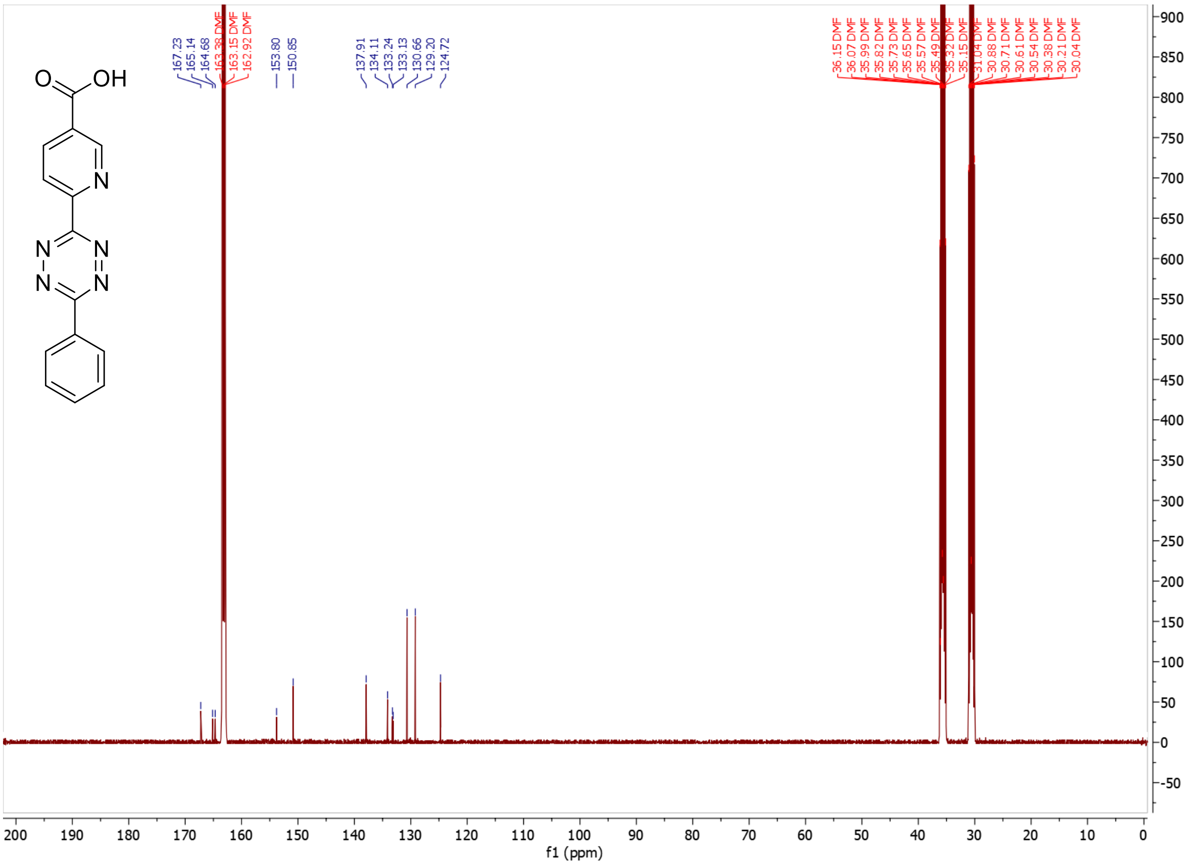

Figure S14. ^{1}H and ^{13}C NMR spectra of compound **18** recorded in DMF-d_7 at 500 MHz and 126 MHz, respectively.

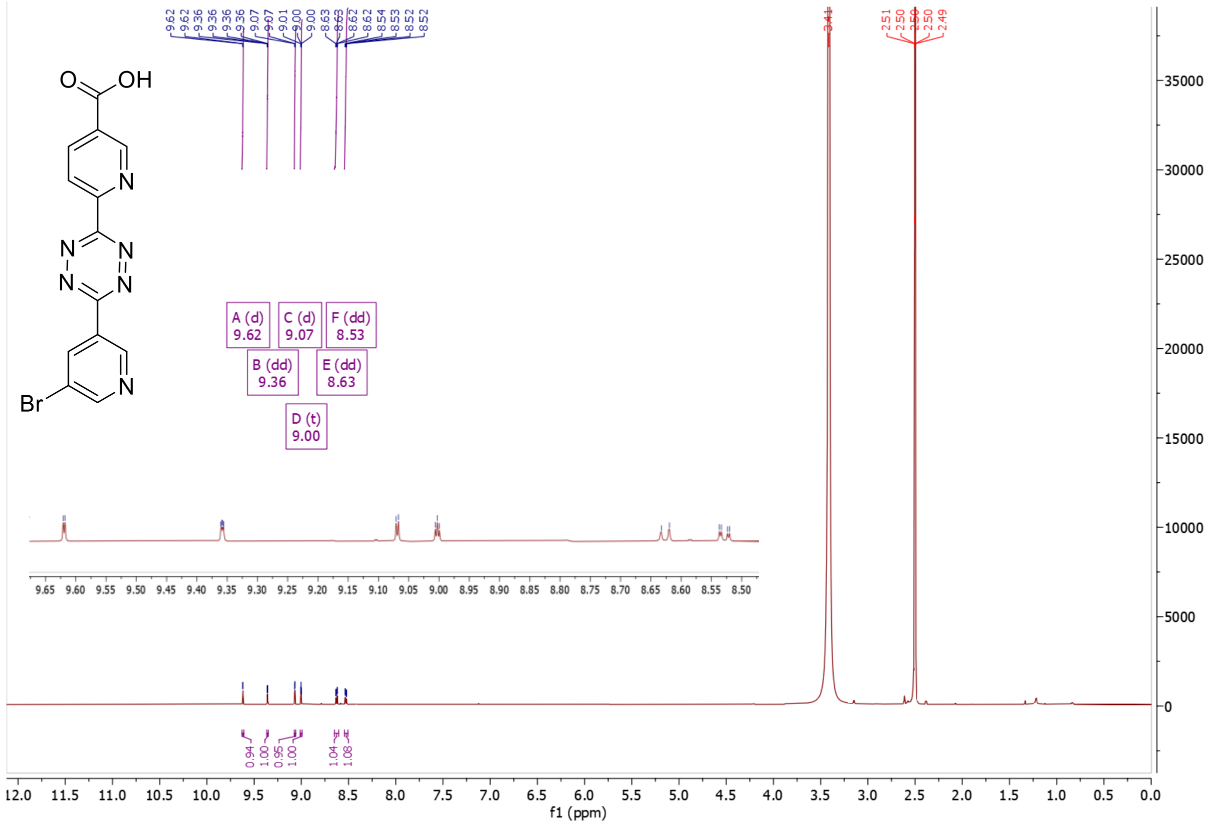

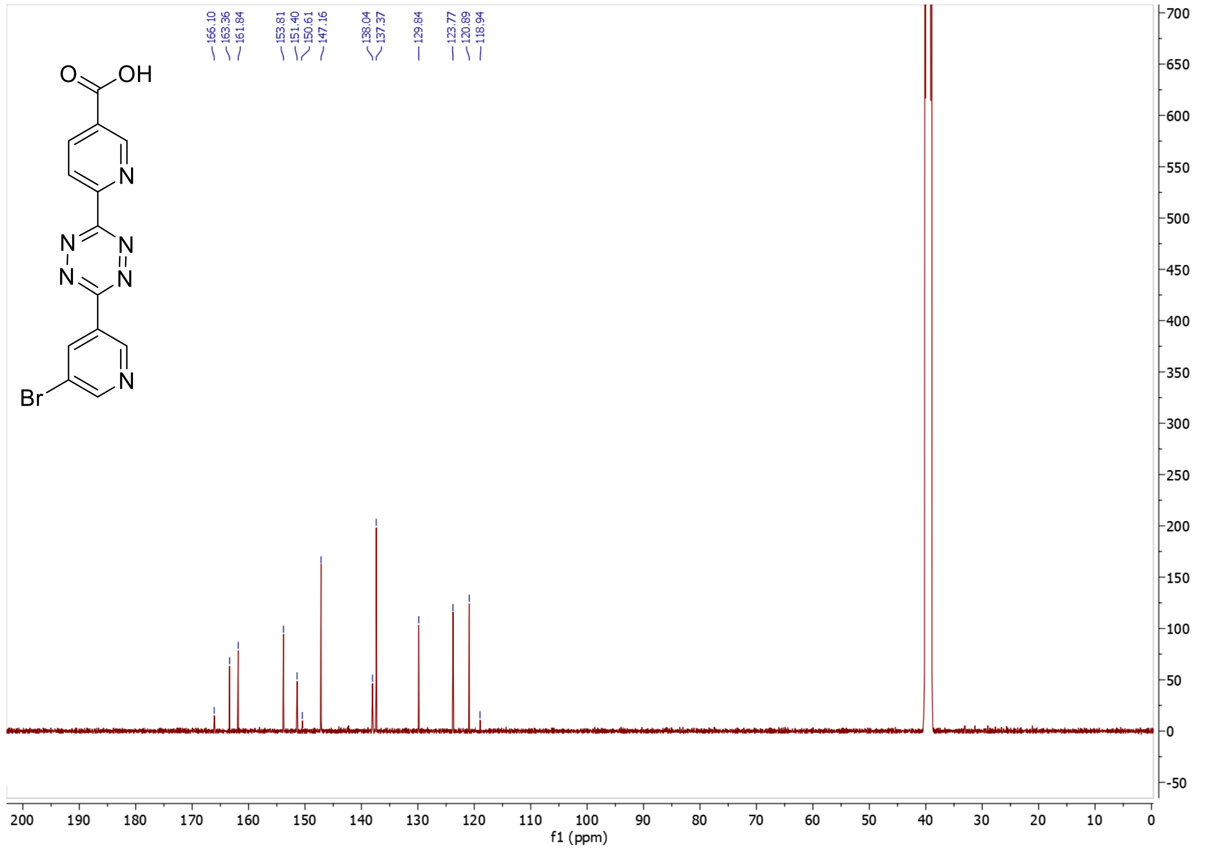

Figure S15. ^{1}H and ^{13}C NMR spectra of compound **19** recorded in DMSO-d_6 at 600 MHz and 126 MHz, respectively.

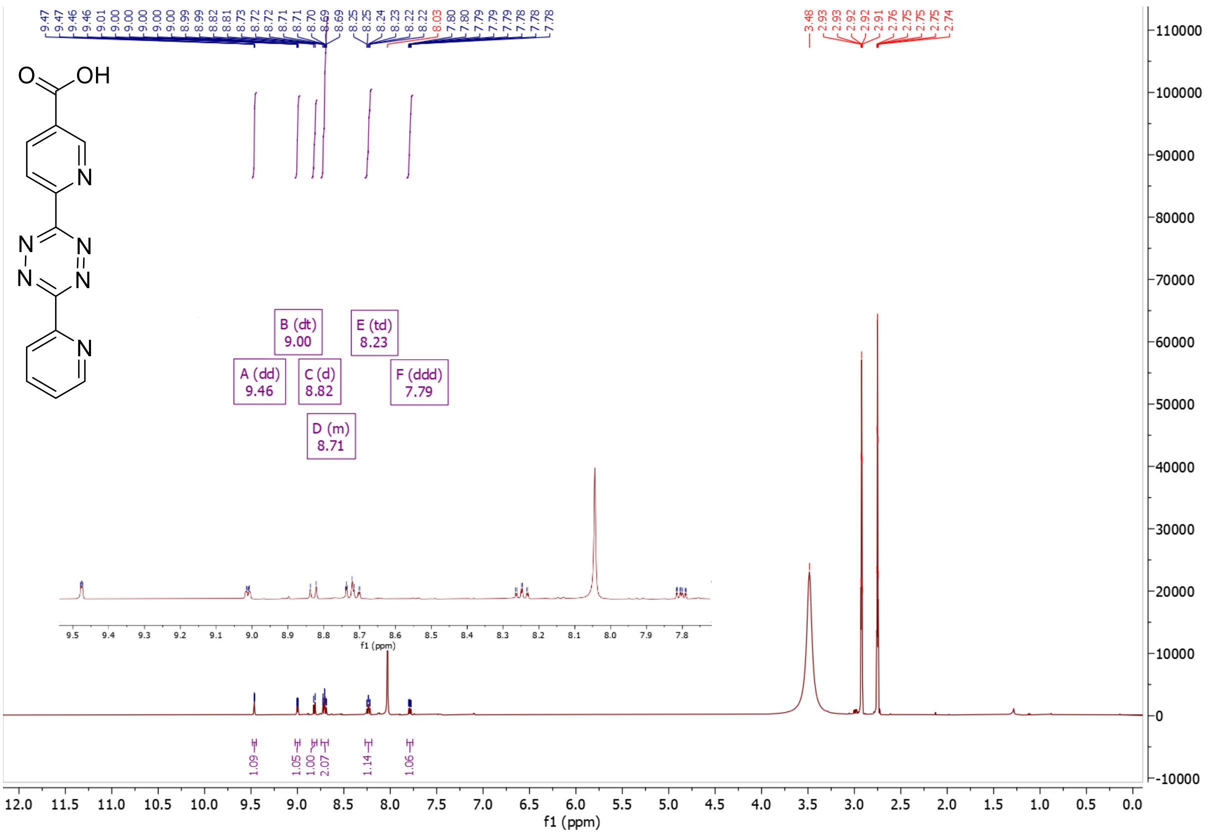

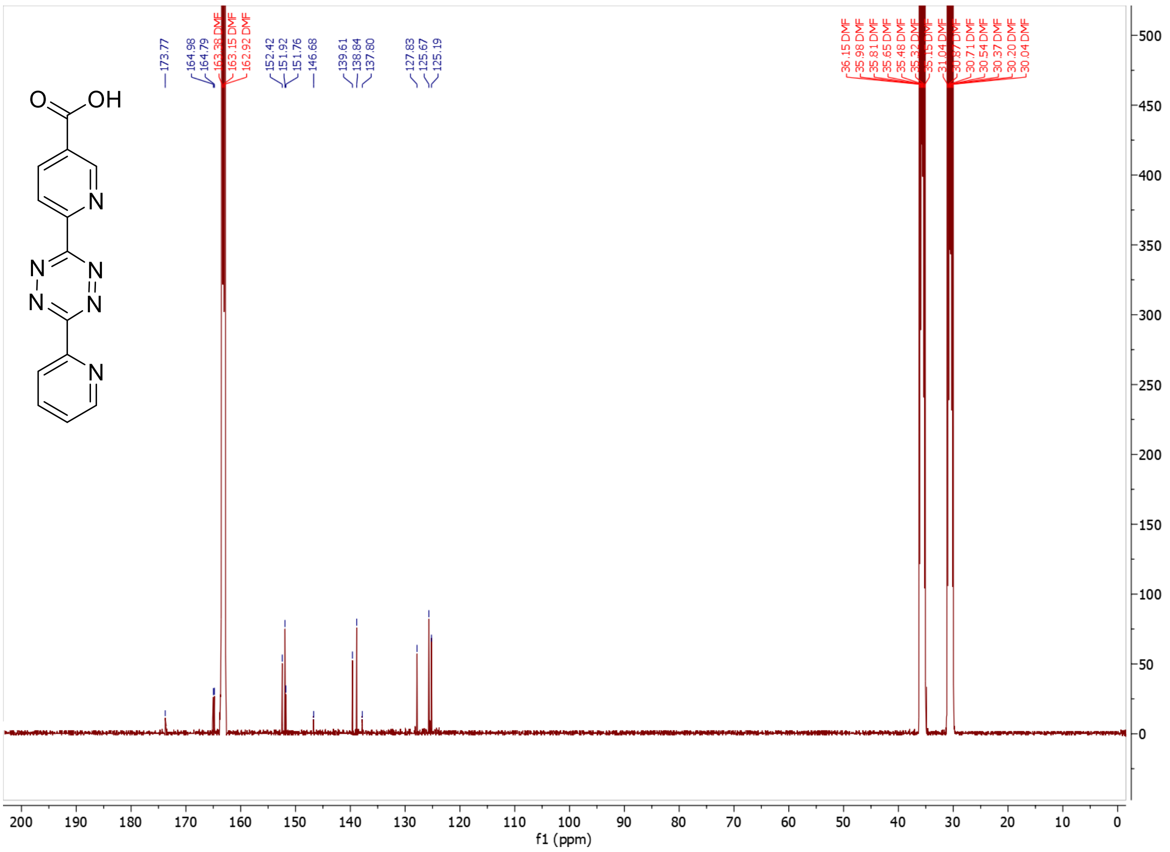

Figure S16. ^{1}H and ^{13}C NMR spectra of compound **20** recorded in DMF-d_7 at 500 MHz and 126 MHz, respectively.

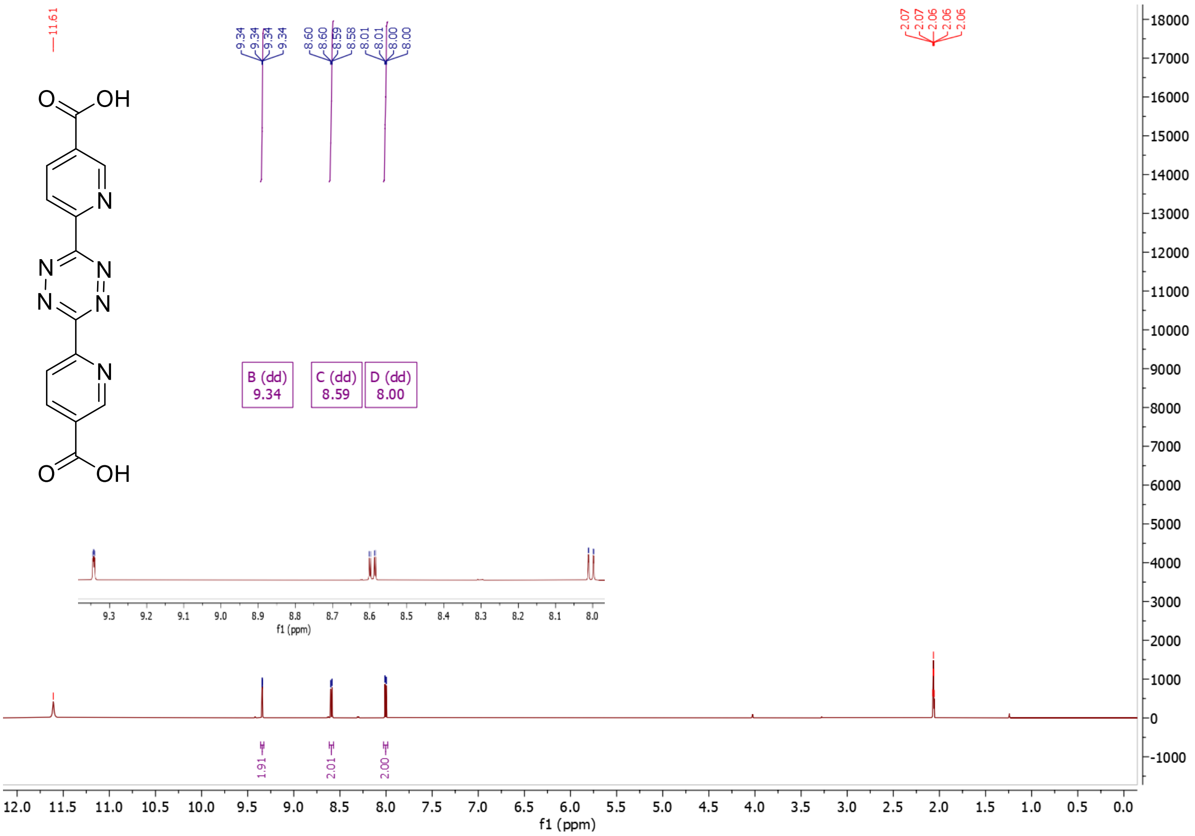

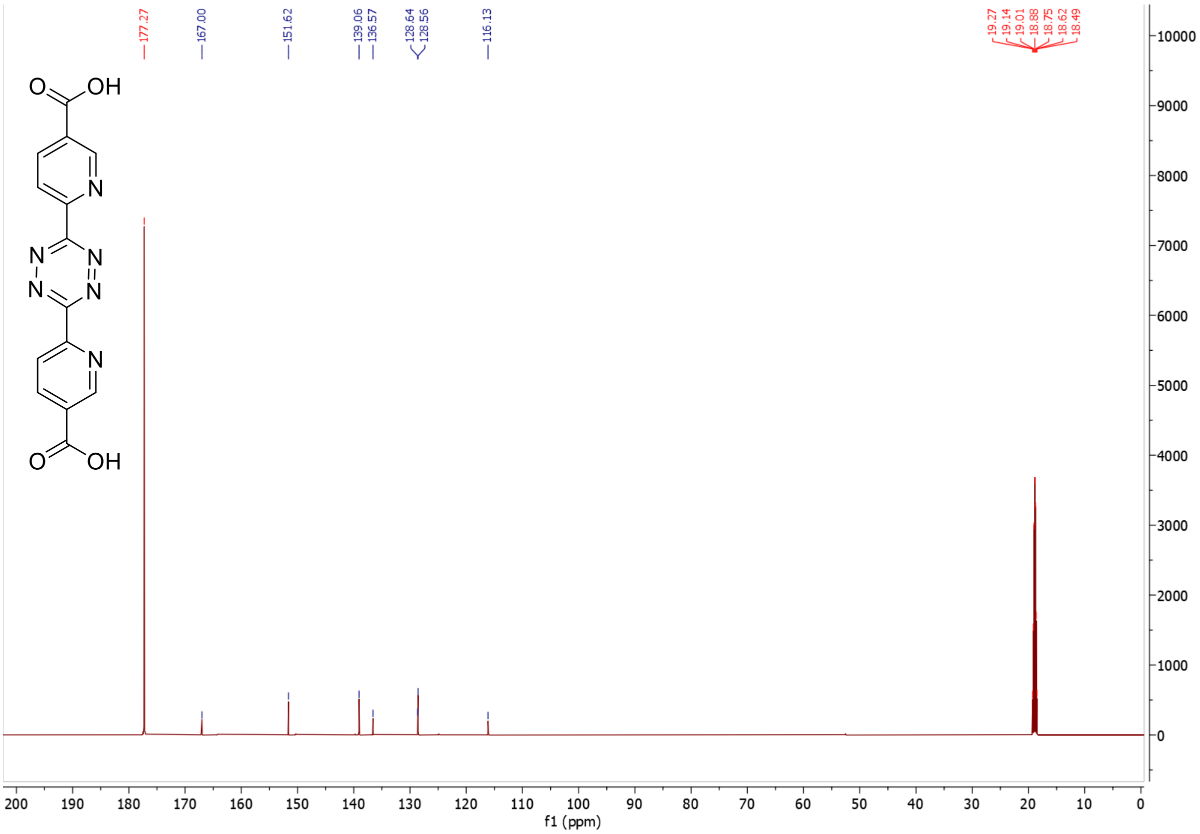

Figure S17. ^{1}H and ^{13}C NMR spectra of compound **21** recorded in acetic acid-*d4* at 600 MHz and 151 MHz, respectively.

5. HRMS

Compound 4

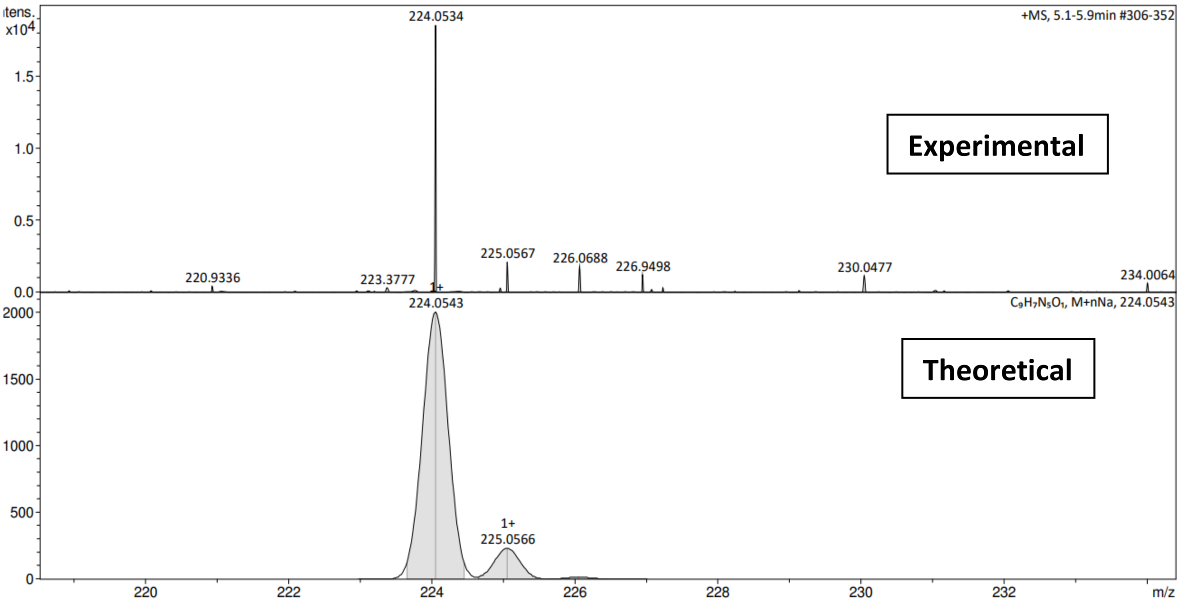

Compound 5

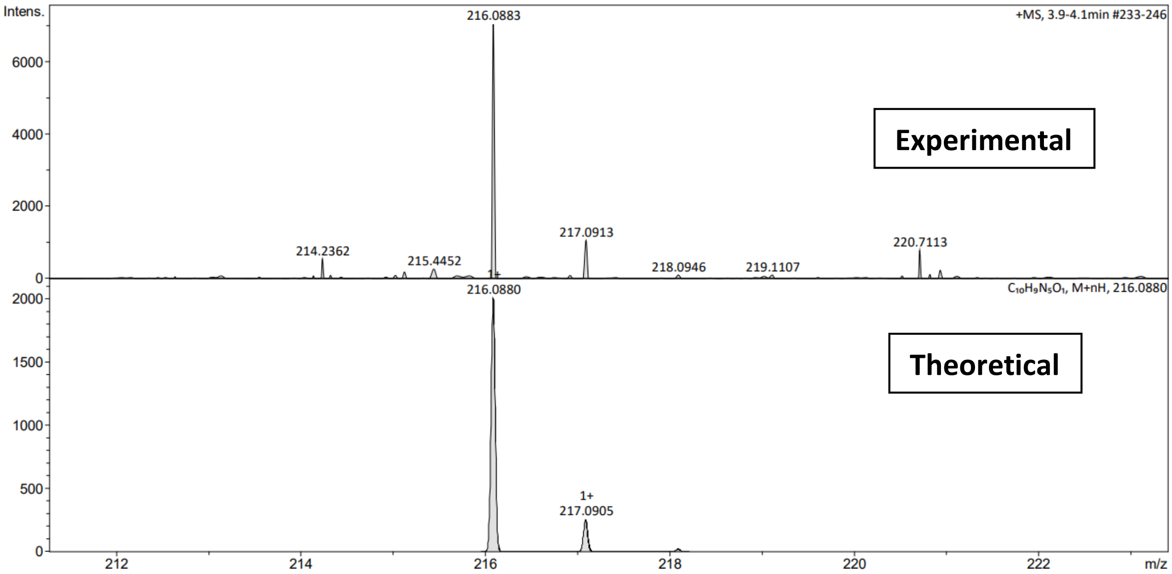

Compound 6

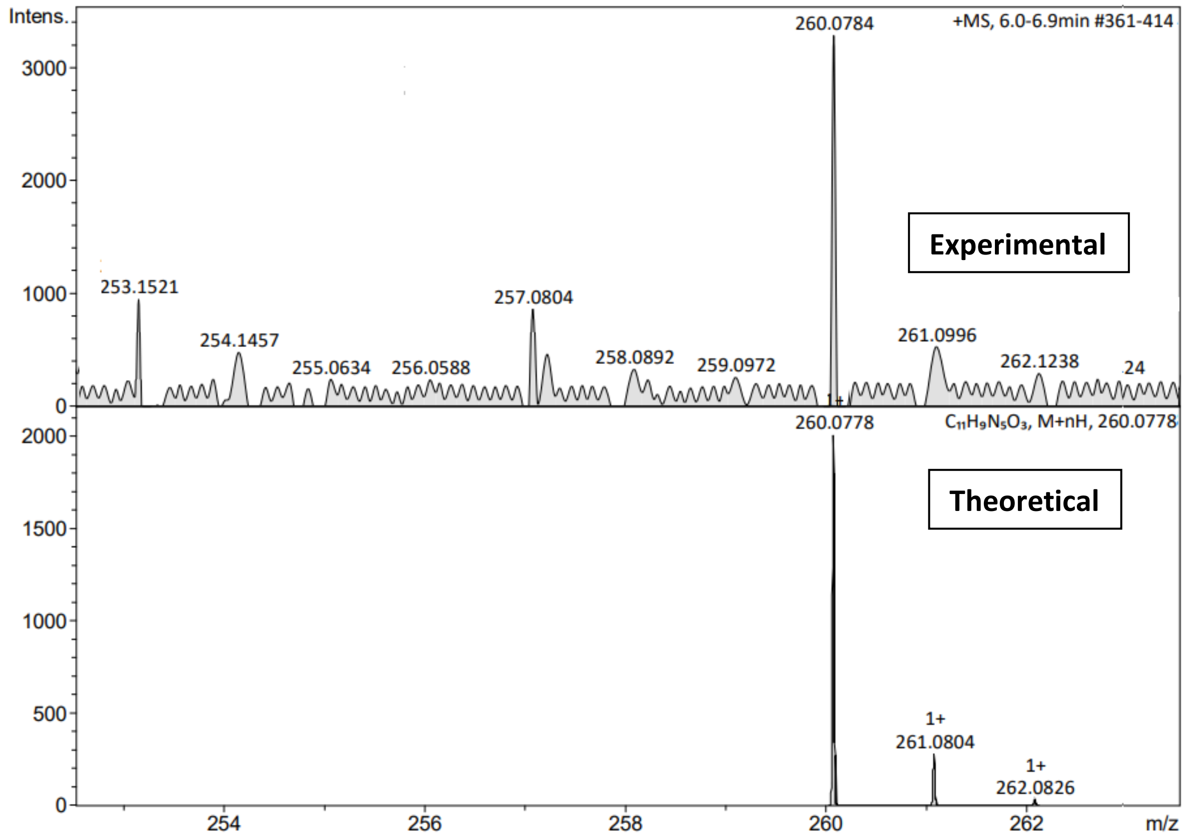

Compound 7

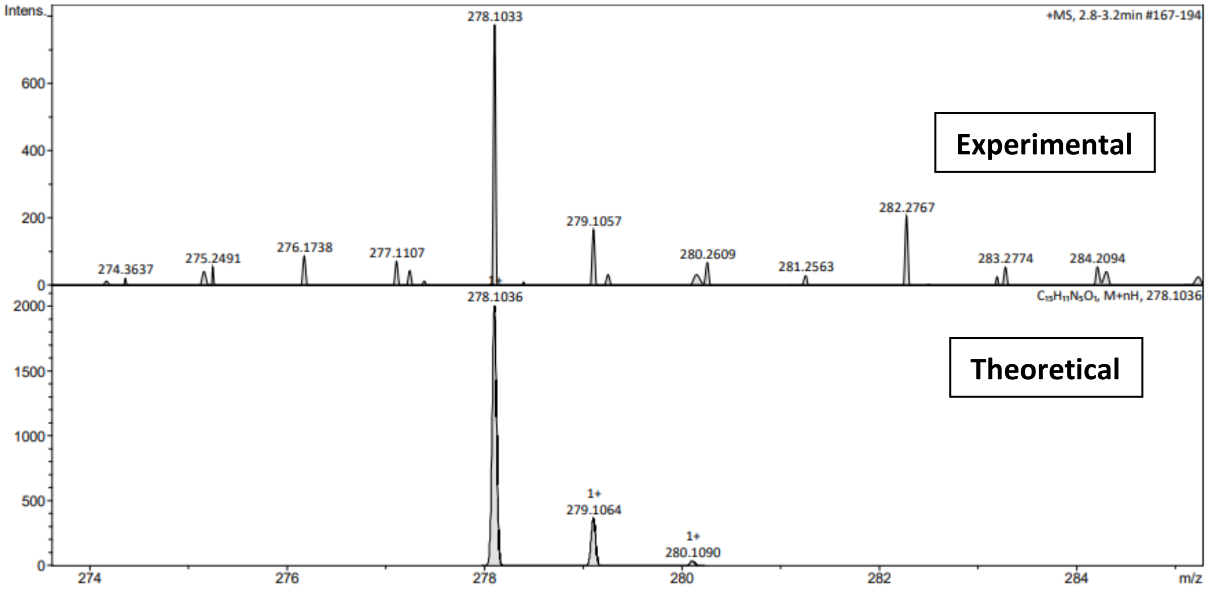

Compound 8

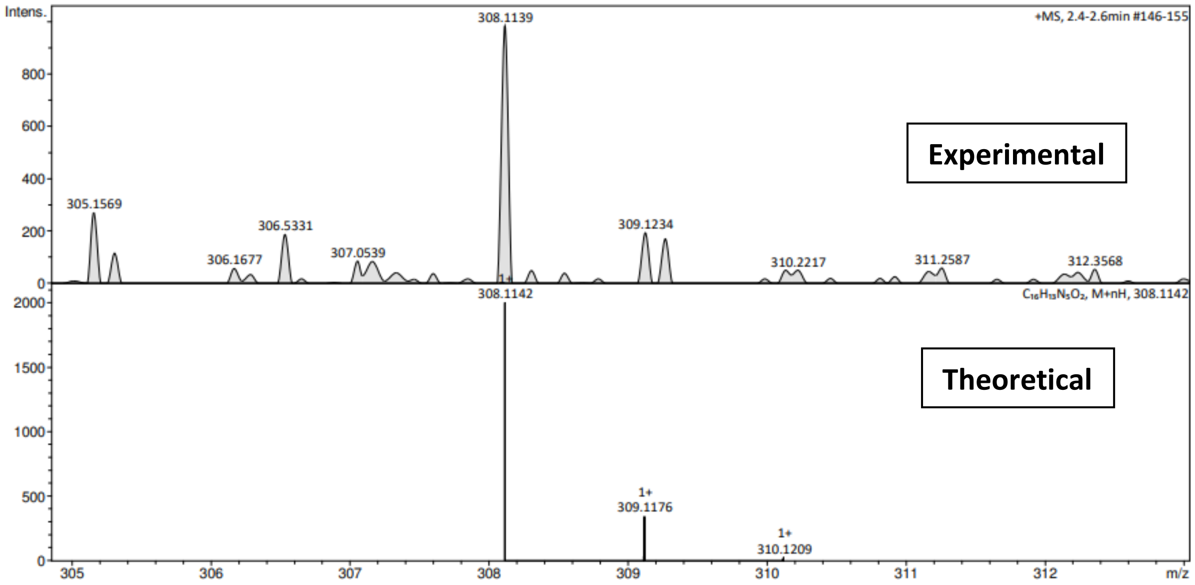

Compound 9

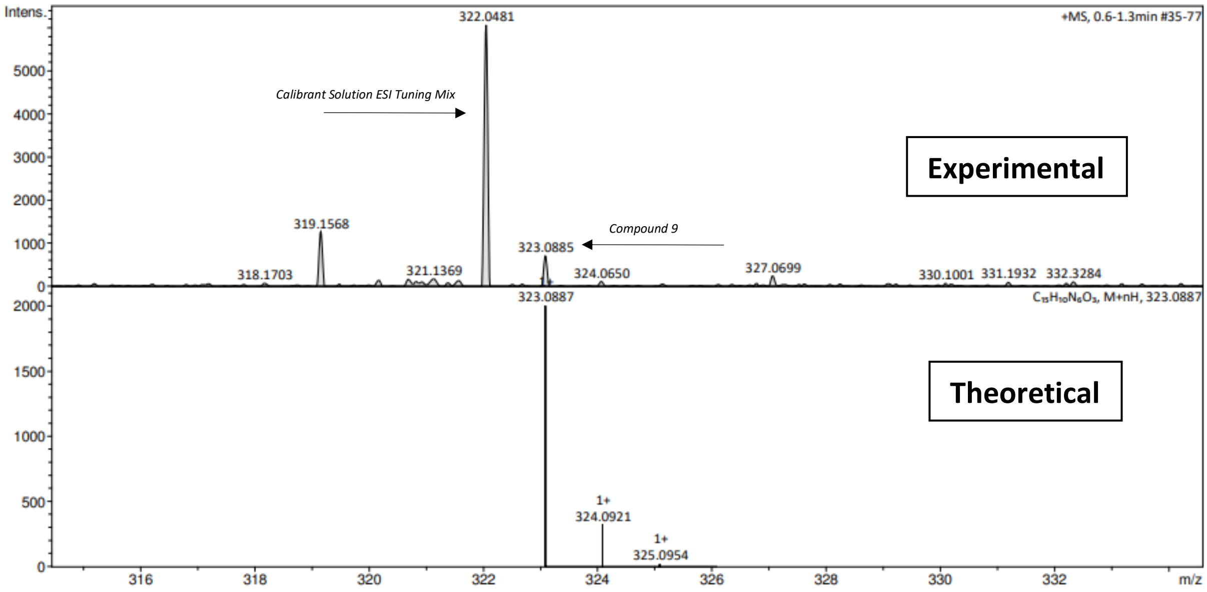

Compound 10

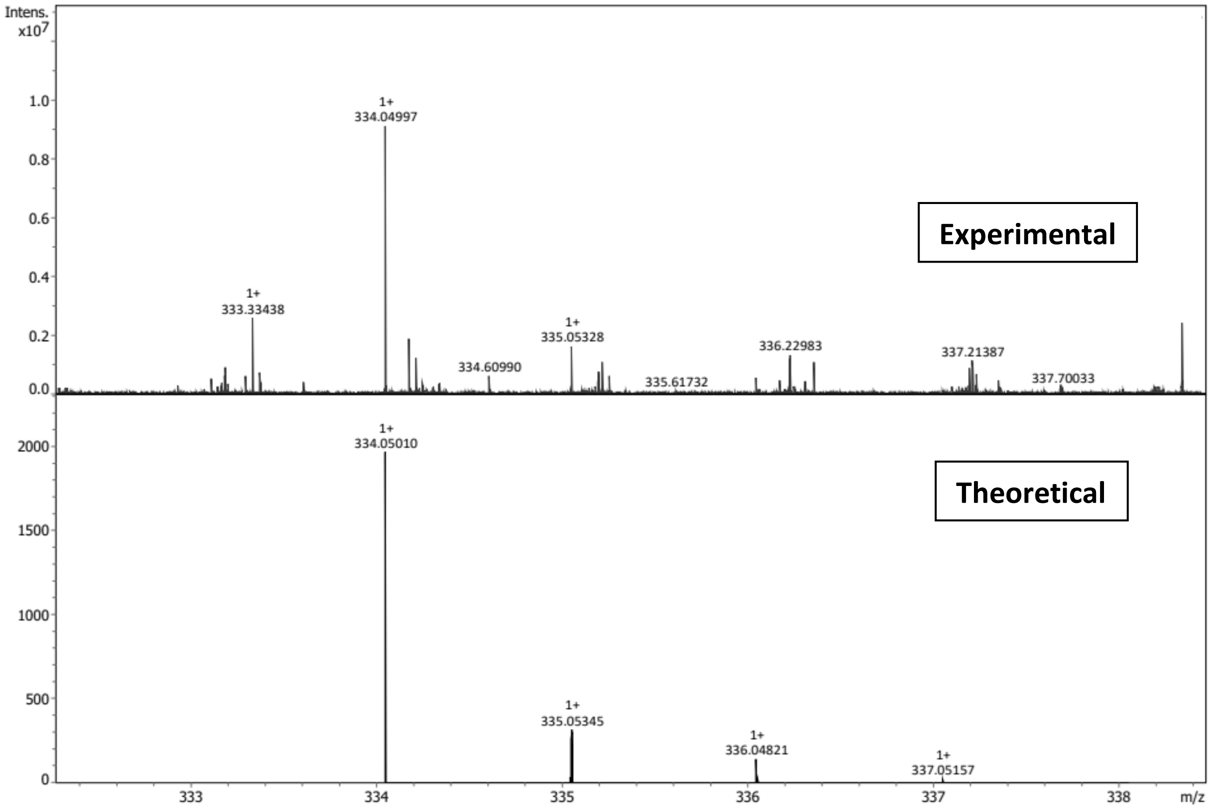

Compound 13

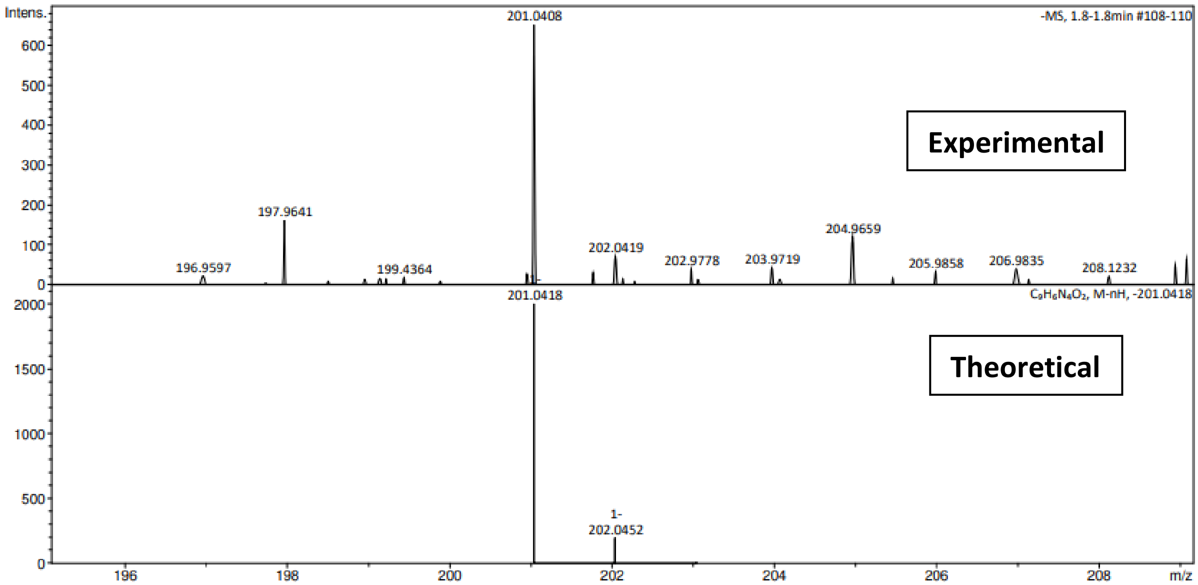

Compound 14

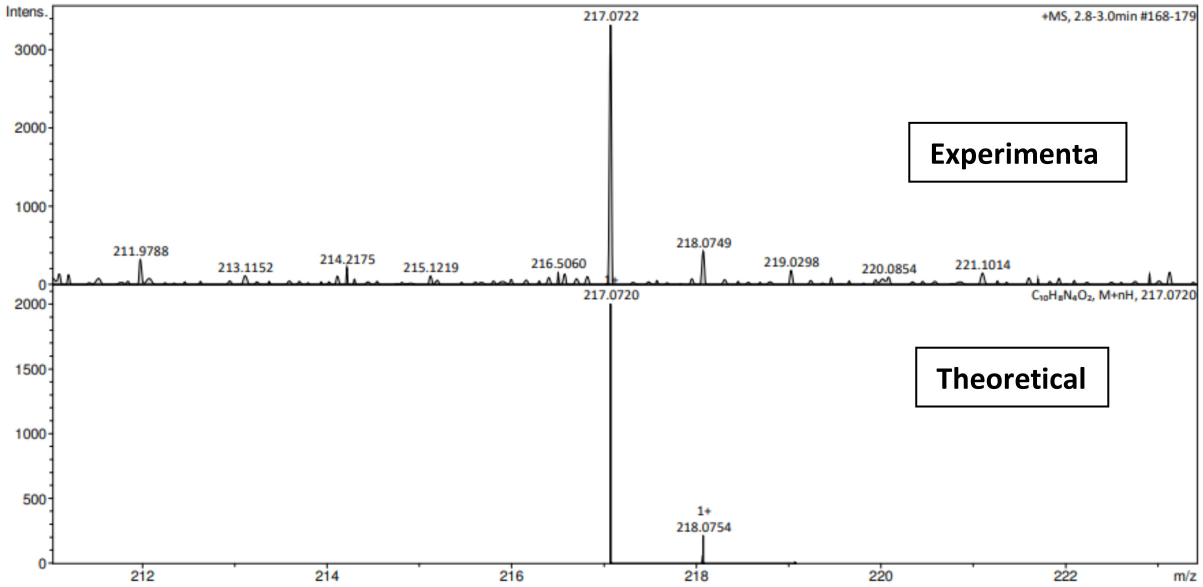

Compound 15

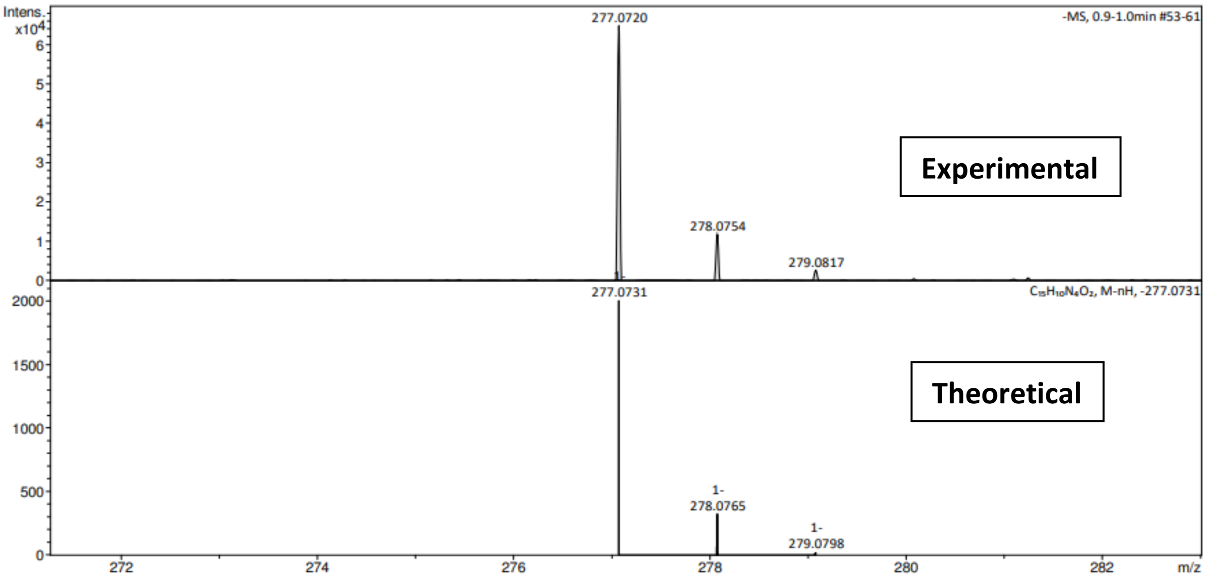

Compound 16

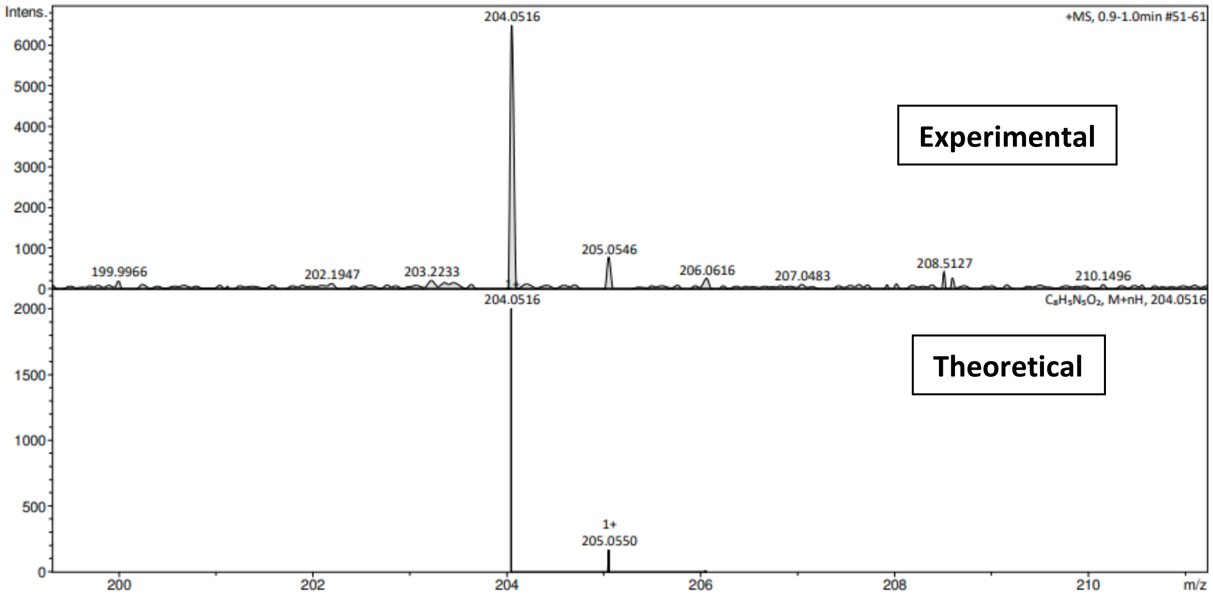

Compound 17

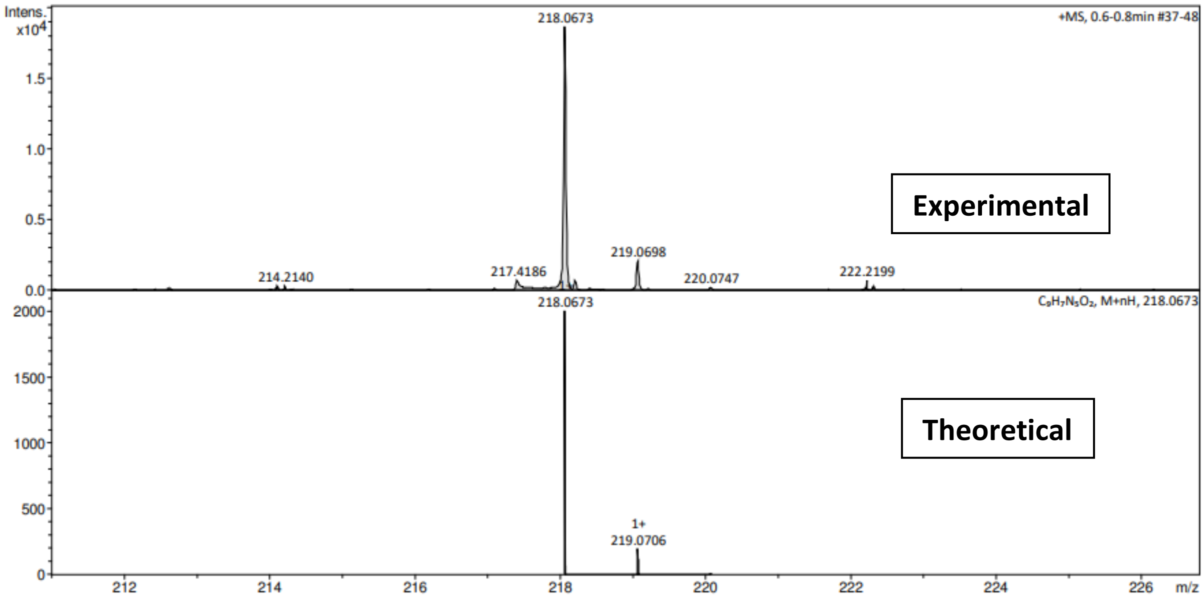

Compound 18

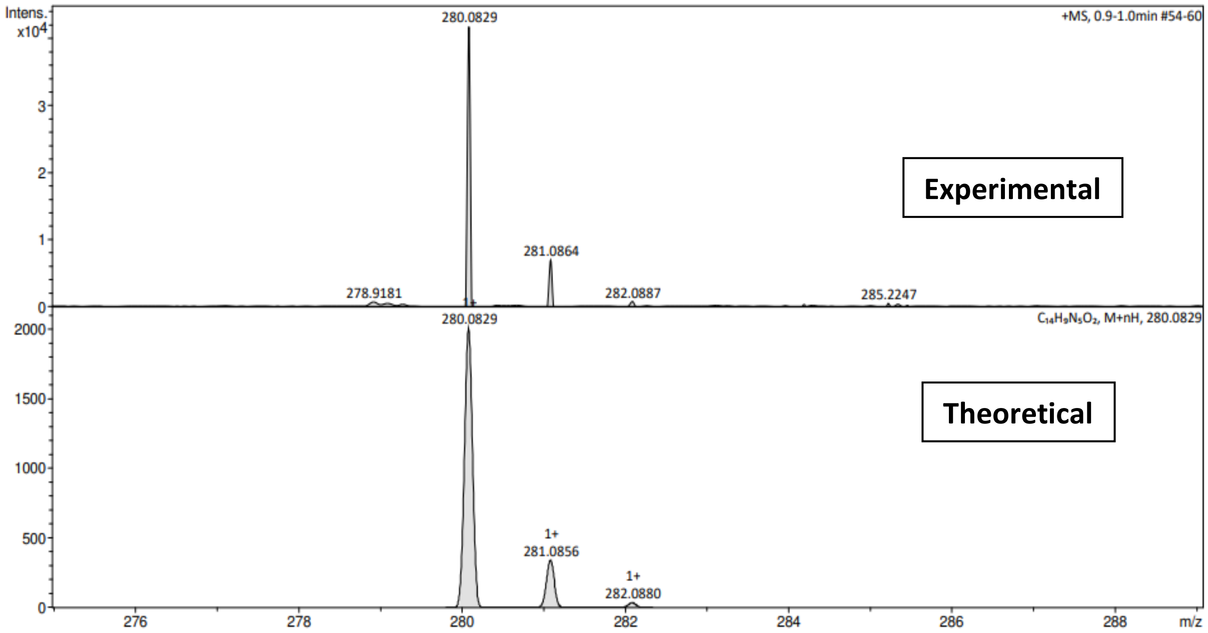

Compound 19

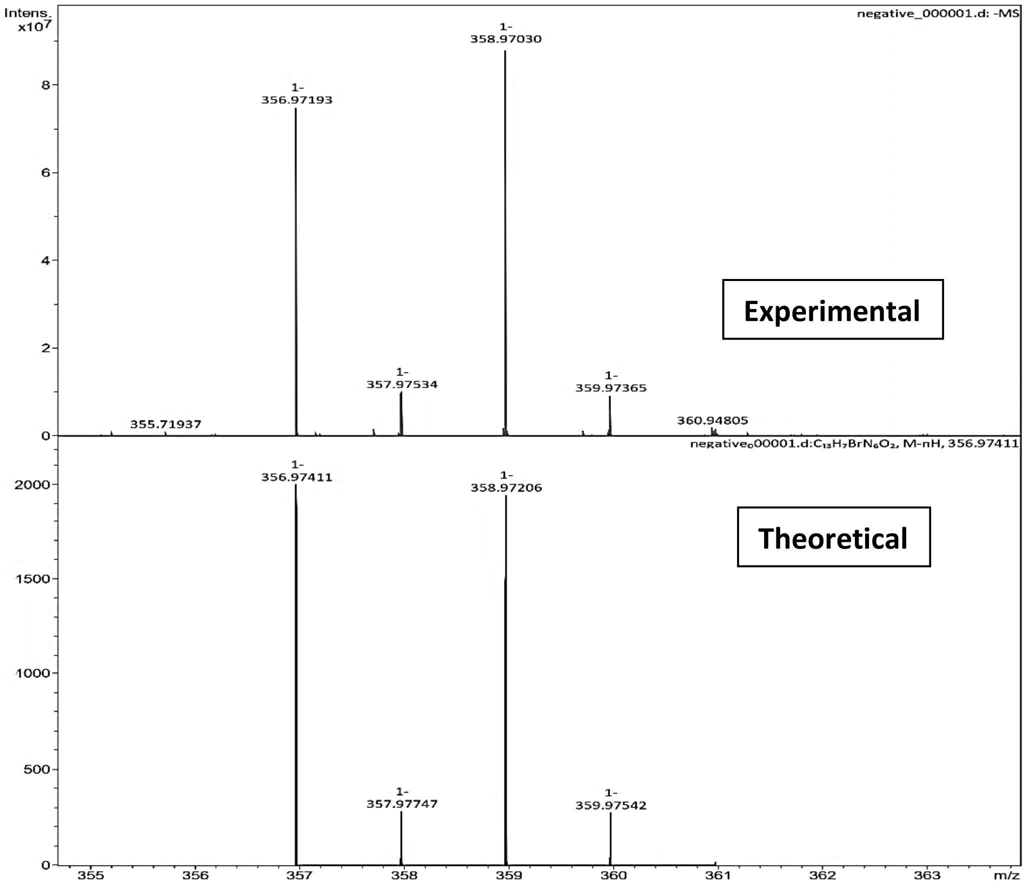

Compound 20

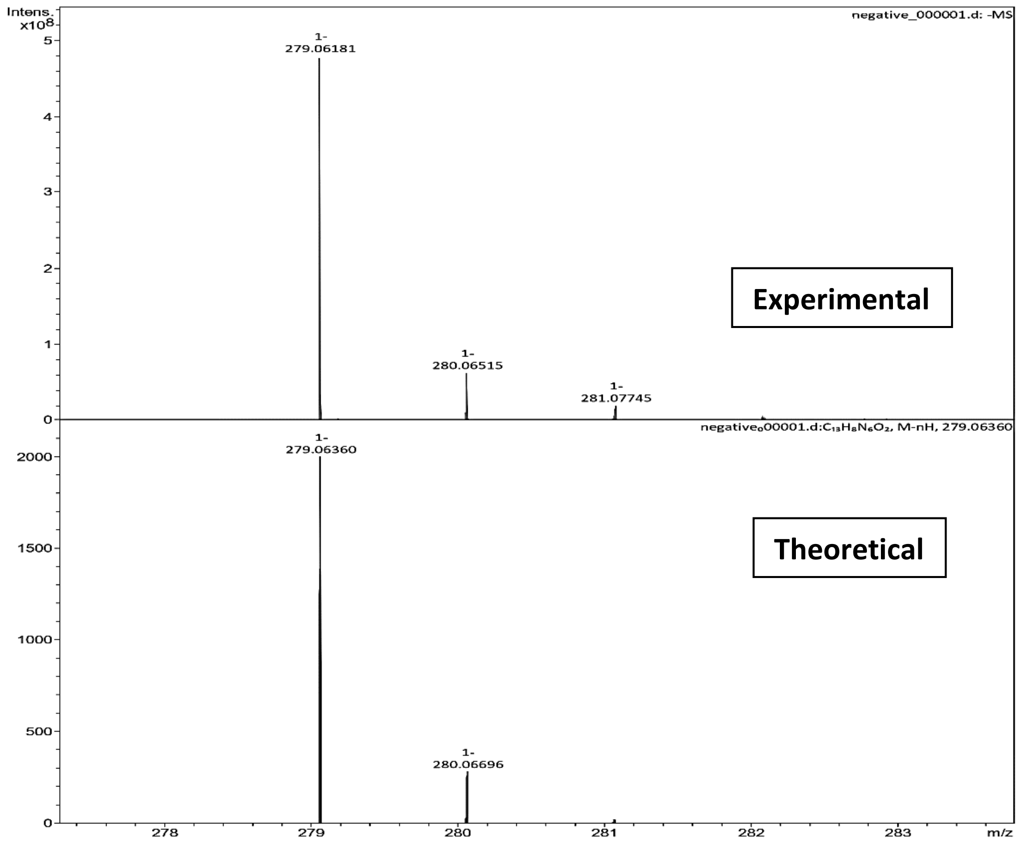

Compound 21

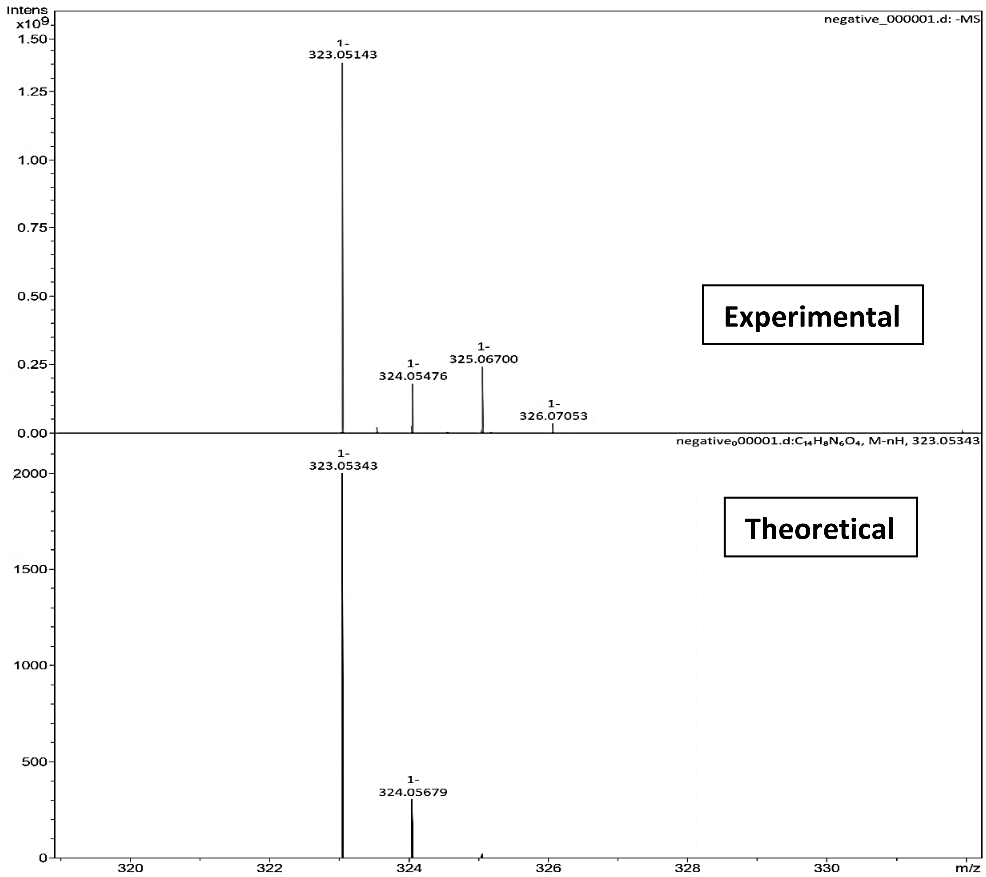

6. References:

(1) Kaiser, E.; Colescott, R. L.; Bossinger, C. D.; Cook, P. Color test for detection of free terminal amino groups in the solid-phase synthesis of peptides. *Analytical biochemistry* **1970**, *34* (2), 595-598.

(2) Devaraj, N. K.; Karver, M. R.; Hilderbrand, S. A.; Weissleder, R. Preparation of functionalized 1,2,4,5-tetrazine compounds for use in bioorthogonal coupling reactions. WO2014065860, **2014**.

(3) Herth, M.; Kjaer, A.; Joergensen, J. T.; Battisti, U. M.; Bratteby, K. E. Aliphatic 18F-radiolabeling of a tetrazine precursor. WO2022189304, **2022**.
